# Genetic Effects and Expression Patterns of the Nitrate Transporter (NRT) Gene Family in *Populus tomentosa*

**DOI:** 10.3389/fpls.2021.661635

**Published:** 2021-05-13

**Authors:** Lei Zhao, Panfei Chen, Peng Liu, Yuepeng Song, Deqiang Zhang

**Affiliations:** ^1^Beijing Advanced Innovation Center for Tree Breeding by Molecular Design, Beijing Forestry University, Beijing, China; ^2^National Engineering Laboratory for Tree Breeding, College of Biological Sciences and Technology, Beijing Forestry University, Beijing, China; ^3^Key Laboratory of Genetics and Breeding in Forest Trees and Ornamental Plants, College of Biological Sciences and Technology, Beijing Forestry University, Beijing, China

**Keywords:** association genetics, expression pattern, leaf nitrogen content, *NRT* gene family, *Populus*, transcription factor

## Abstract

Nitrate is an important source of nitrogen for poplar trees. The nitrate transporter (*NRT*) gene family is generally responsible for nitrate absorption and distribution. However, few analyses of the genetic effects and expression patterns of *NRT* family members have been conducted in woody plants. Here, using poplar as a model, we identified and characterized 98 members of the *PtoNRT* gene family. We calculated the phylogenetic and evolutionary relationships of the *PtoNRT* family and identified poplar-specific *NRT* genes and their expression patterns. To construct a core triple genetic network (association - gene expression - phenotype) for leaf nitrogen content, a candidate gene family association study, weighted gene co-expression network analysis (WGCNA), and mapping of expression quantitative trait nucleotides (eQTNs) were combined, using data from 435 unrelated *Populus*. *tomentosa* individuals. *PtoNRT* genes exhibited distinct expression patterns between twelve tissues, circadian rhythm points, and stress responses. The association study showed that genotype combinations of allelic variations of three *PtoNRT* genes had a strong effect on leaf nitrogen content. WGCNA produced two co-expression modules containing *PtoNRT* genes. We also found that four *PtoNRT* genes defined thousands of eQTL signals. WGCNA and eQTL provided comprehensive analysis of poplar nitrogen-related regulatory factors, including MYB17 and WRKY21. *NRT* genes were found to be regulated by five plant hormones, among which abscisic acid was the main regulator. Our study provides new insights into the *NRT* gene family in poplar and enables the exploitation of novel genetic factors to improve the nitrate use efficiency of trees.

## Introduction

Nutrient absorption, transport and recycling are key processes in the plant life cycle. During seasonal leaf senescence, trees reabsorb specific nutrients from the leaves. These nutrients are stored in stems and roots and used at the beginning of the next growing season to support new growth (Babst and Coleman, 2018). Seasonal nutrient storage gives long-lived perennials an advantage over other plants. Nitrogen is one of the macronutrients necessary for the growth of forest trees. It is a major component of essential compounds such as amino acids, nucleotides, chlorophyll, hormones and vitamins (Xu et al., 2012). N availability is usually the main factor limiting the productivity of trees. The nitrogen use efficiency (NUE) of plants is defined as the efficiency with which plants obtain and use nitrogen. Transporters play a key role in nitrogen-related signaling, metabolism, and physiology, as they allow the movement of water and solutes (such as inorganic ions, hormones, amino acids, and sugars) across biological membranes (Hedrich, 2012). Nitrate (NO_3_^–^) is the main N source used by higher plants. Most NO_3_^–^ obtained by plants from soil is actively transported by a NO_3_^–^ transporter (NRT) (Gojon et al., 2011). To adapt to fluctuating nitrate concentrations in soil, plant roots have developed a low-affinity transport system (LATS, >1 mM) and high-affinity transport system (HATS, 1 μM ∼ 1 mM). According to whether the specific soil nitrate concentration could induce gene expression, two types of transport modes were differentiated, namely constitutive (cLATS/cHATS) and inducible (iLATS/iHATS) (O’Brien et al., 2016).

*AtNRT1.1* (also known as *CHL1*/*NPF6.3*) of *Arabidopsis thaliana* was the first member of the NRT family to be identified (Tsay et al., 1993) and has dual affinities for nitrates (Liu et al., 1999). This dual-affinity nitrate transporter is involved in nitrate absorption and root-to-shoot transport, and also plays an important role in nitrate-induced auxin transport and regulation of root morphology (Bouguyon et al., 2015). In *A. thaliana*, NRT1.5 is a low-affinity bidirectional nitrate transporter that participates in the loading of nitrate into the root xylem, which is essential for the transport and outflow of nitrate nitrogen from root to stem (Lin et al., 2008). AtNRT1.6 is mainly responsible for delivering nitrate from maternal tissues to developing embryos (Almagro et al., 2008), which also suppresses NO_3_^–^ starvation-induced leaf senescence (Meng et al., 2016). The function of AtNRT1.8 is absorbing nitrate into xylem parenchyma cells, thereby removing nitrate from the xylem sap (Li et al., 2010). AtNRT1.9 promotes the loading of nitrate into the root phloem and enhances the downward transport of nitrate within roots (Wang and Tsay, 2011). The *NRT1* subfamily genes were revised nomenclature for the characterized *NPF* members (Leran et al., 2014). The NRT2 subfamily is a high-affinity nitrate transporter. NAR2, which forms a complex with NTR2 (Kotur et al., 2012), is named NRT3.

Arabidopsis and rice contain 53 and 93 *NPF* genes, respectively. The functions of many *NRT* genes remain unknown, and only a few have been characterized. *OsNRT1*/*NPF8.9* was the first *NRT* gene identified in rice (Lin et al., 2000). The spatial expression pattern of *OsNRT1* suggests that it may be involved in nitrate absorption. *OsNRT1.1b*/*OsNPF6.5* is one of the closest linear homologs of *AtNPF6.3* in rice, and it also encodes a nitrate transporter that regulates nitrate absorption and root-stem transport (Hu et al., 2015). Interestingly, a single nucleotide polymorphism (SNP) leading to Thr327Met substitution between *indica* and *japonica* varieties is responsible for the enhanced nitrate uptake, root-stem transport, nitrate assimilation, and nitrogen use efficiency (NUE) of *indica* rice. These results indicate that marker-assisted molecular breeding of improved nitrate transporters is a feasible method for improving plant NUE. In rice, OsNPF7.3/PT6 mediates peptide transport and plays roles in regulating total nitrogen content and plant growth (Fang et al., 2017). Overexpression of transporters in the *NRT1*(*NPF*) and *NRT2* subfamilies can also enhance NUE. For example, overexpression of rice *OsNRT2.3b* not only increased nitrate and iron uptake but also increased yield under both low and high-nitrogen field conditions (Fan et al., 2016). A specific promoter is necessary for some transporters to improve NUE of transgenic plants. Transporters are generally expressed specifically in certain tissues or cells. When they are constitutively and universally expressed, they have negative impacts on NUE and yield. For example, the introduction of ubiquitin-driven *OsNRT2.1* into rice resulted in decreased NUE and grain yield, whereas the introduction of the nitrate-induced promoter *OsNAR2.1p* had the opposite effect (Chen et al., 2016).

Poplars are perennial deciduous woody plants. Their NUE can be effectively improved by increasing the absorption capacity of nitrate in roots and the redistribution capacity of nitrate in other tissues. The expression pattern of *NRT* gene family members shows obvious tissue specificity, but few tissues have been subjected to tissue-specific analysis (Bai et al., 2013), especially the xylem, cambium, phloem and other tissues peculiar to woody plants. Due to the large number of *NRT* gene family members, the genetic effects of *NRT* genes on the nitrogen content and growth traits of poplar remain unclear. Therefore, we carried out relevant research on an important plantation tree species in China, *P*. *tomentosa*, to elucidate nitrogen utilization associated with the *NRT* gene family in poplar. We examined population expression data and comprehensive tissue-specific data for *P. tomentosa* and preliminarily analyzed the correlations between genotype, expression and leaf nitrogen content through association analysis. Our study provides new insights into the genetic regulation of perennial tree nutrition and growth traits.

## Materials and Methods

### Plant Materials and Growth Conditions

As described in a previous manuscript, a 10-year-old association population of 435 unrelated individuals was vegetatively propagated in Guanxian County, Shandong Province, China (36° 23′N, 115° 47′E) in 2009, from root segments, using a random complete block design of three blocks (Xiao et al., 2020). The sampled population was randomly selected from 1047 individuals from natural *P. tomentosa* populations, representing almost the entire natural distribution of *P. tomentosa* (30-40° N, 105-125° E), which can be divided into three climatic regions (Zhang et al., 2010). The DNeasy Plant Mini kit (Qiagen, Shanghai, China) was used to extract total genomic DNA from fresh leaves of each individual according to the manufacturer’s protocol.

### Identification of *NRT* Genes in *P. tomentosa*

The whole genome of *P. tomentosa* was sequenced using the Pacbio-HiFi method. All known 62 *NRT* gene sequences of *Arabidopsis thaliana* were downloaded from the AtGenIE^1^. The protein sequences of 62 *AtNRT* genes were used as queries for the Basic Local Alignment Search Tool (BLAST) against the *P. tomentosa* genome. The identified PtoNRT protein sequences were uploaded to the National Center for Biotechnology Information (NCBI) Protein BLAST program (blastp^2^) for comparison against the *Arabidopsis* genome using the UniProtKB/Swiss-Prot database. This process resulted in exact matches of 62 *Arabidopsis NRT* genes. All candidate PtoNRT protein sequences were further screened based on their conserved domains (CDs) using the NCBI Batch CD Search program^3^. The observed CDs included those of major facilitator superfamily (MFS), nitrate reductase (NAR) and phospholamban (PLN) proteins, as expected in Arabidopsis. We obtained homologous *PtoNRT* genes from *P. trichocarpa* using the BLAST to uniform gene ID. The *cis*-regulatory elements (CREs) for the promoter sequence of *NRT* family genes in *P*. *tomentosa* were predicted using plantCARE^4^. The CDS and protein sequences of *PtoNRT* genes were uploaded to GenBank Banklt (accession numbers: MW544773 - MW544870)^5^.

### Bioinformatic and Phylogenetic Analysis

The isoelectric point (pI) and molecular weight of PtoNRT protein were predicted using ExPASy^6^. Identified PtoNRT protein sequences were subjected to multiple sequence alignment in MEGA X software^7^. For treatment of gaps and missing data, we selected partial deletion with a site coverage cutoff of 80%. The optimal amino acid substitution model was identified as Jones-Taylor-Thornton (JTT) + (G) + (F). A phylogenetic tree of protein sequences was constructed using the maximum likelihood (ML) approach with 1000 bootstrap replicates in MEGA X. All positions with less than 90% site coverage were eliminated, or in other words, less than 10% total alignment gaps, missing data, and ambiguous bases were allowed for any position. Figtree^8^ was used to visualize the phylogenetic tree.

### Gene Structure, Conserved Motif, and Chromosomal Mapping Analysis

The Multiple Expectation maximizations for Motif Elicitation (MEME) program^9^ was used to analyze conserved motifs in PtoNRT protein sequences. Gene structures, conserved motifs and domains within the phylogenetic tree were visualized using TBtools (Chen et al., 2020), which need to provide tree file, MAST.XML file, domain file, and GFF3 file of *P. tomentosa*. Chromosomal positioning was determined in TBtools using the GFF3 file and a gene list.

### Collinearity Analysis

The whole full length protein sequences from *P. tomentosa* were aligned with themselves using BLAST with a cut-off e-value of 10^–5^. Collinearity blocks across the entire genome and collinear pairs between PtoNRT proteins were located using MCScanX software according to the blastp results. From the collinearity file, tandem file and gene list file, visualization of the collinearity map was conducted in TBtools. The blastp results were also used to calculate the K_*a*_/K_*s*_ ratio for *P. tomentosa*. To analyze collinearity between *Arabidopsis thaliana* and *P. tomentosa*, the entire protein sequences of the species were aligned. The two BLAST files and two GFF3 files for the two species were integrated using TBtools, and then the collinearity file was created in MCscanX. Finally, a dual systeny plot was produced in TBtools using the GFF3 file, collinearity file, gene list file and a control file.

### RNA-seq Analysis and Gene Expression Heatmap

Tissue-specific sampling included samples of young leaf, expanded leaf, old leaf, apex, root, mature xylem, immature xylem, cambium, phloem, bark, petiole, pistil, stamen, and leaves on long and short branches. All samples were taken from the 1-year-old *P. tomentosa* clone “LM50,” planted in Guanxian County, and promptly placed into liquid nitrogen. Leaves of LM50 grown in a growth cabinet (14-h light, 10-h dark, 28°C) were used as sample for circadian rhythm and stress treatment. Circadian rhythm samples were taken every 2 h over 24 h. Samples from the ABA, drought, heavy metal, high-salt, and high-temperature treatments were taken at 1, 3, 6, 12, and 18 h. We also collected leaves from the *P. tomentosa* population as samples. Each of the samples described above was analyzed in three biological replicates. All transcriptome data used in this study are provided in Supplementary Datasheet 1. Statistical analysis was performed using analysis of variance (ANOVA) in the R 3.6.3 to evaluate differentially expressed genes. All transcriptome data have been uploaded to the public database. The transcriptome expression data (three biological replicates per group) are available in the National Center for Biotechnology Information SRA database under accession numbers PRJNA521819, PRJNA521855, PRJNA522886, PRJNA522891, PRJNA357670, SRP141094, SRP073689, and SRP060593.

Total RNAs were extracted using the Plant Qiagen RNAeasy kit following the manufacturer’s instructions. Total RNAs were used for transcriptome sequencing. The FPKM (fragments per kilobase of transcript per million fragments) method was used to normalize transcript expression. The processing of transcriptome data was described in a previous manuscript (Quan et al., 2019).

### Determination of Nitrogen Content in Leaves

We collected old leaves from the *P. tomentosa* population (Guanxian County) in both summer and autumn. We used an oven to remove moisture from the leaves. The dry leaves were then ground into a powder. We accurately weighed a sample of 0.2500-0.5000 g (accurate to 0.0001 g), placed it in a polytetrafluoroethylene digestion tank, added 5 mL nitric acid (superior grade purity) and, after a short incubation, placed it in the digestion furnace at 100°C (rising 5°C per minute) until the sample was completely digested. Then, 3 ml of hydrogen peroxide (premium grade purity) was added, and the acid was driven off at 100°C until the volume of the digestion solution was less than 2 ml, which was transferred to a 50 ml plastic volumetric flask and brought up to that volume. An inductively coupled plasma mass spectrometer ICP-MS (model: Agilent 7700x, American Agilent Technologies) was used to determine total elemental contents except mercury. All samples were analyzed in three biological replicates. Leaf nitrogen content results showed a normal distribution (Supplementary Table 1).

### Weighted Gene Co-expression Network Analysis of the *P. tomentosa* Population

Total RNA extracted from the old leaves of 435 unrelated individuals of *P*. *tomentosa* was used for RNA-seq in 2016, following the methods described above. Library construction and sequencing were performed by Beijing Biomarker Technology Corporation (Beijing, China).

We performed WGCNA using the nitrogen contents of leaves in summer and autumn as phenotypes, based on the expression levels from 89 *P*. *tomentosa* individuals (core population) for 7636 genes. The R 3.6.3 (R Core Team, 2020) WGCNA package was used to construct the co-expression network. The correlations between the modules and leaf nitrogen content were represented with R values. The processing of WGCNA data is described in Supplementary Method 1.

### SNP-Based Association Mapping

The resequencing data and methods used for 435 unrelated *P*. *tomentosa* individuals were described in previous study (Quan et al., 2019). We used the mixed linear model (MLM) in TASSEL v5.0 (Bradbury et al., 2007) to test for statistical associations between SNPs and leaf nitrogen content traits in the association population. The K matrix was calculated previously (Du et al., 2012), and the Q matrix was assessed using STRUCTURE v2.3.4 (Evanno et al., 2005) based on three significant subpopulations. The *P*-value was evaluated for each association, and the significance was defined based on *P*-value ≤ 10E-04. We used the EPISNP1 package in epiSNP software (Ma et al., 2008) to test for pairwise epistatic effects. The two-locus interaction effects were divided into groups based on additive (A) and dominance (D) interactions, designated AA, AD, DA, and DD.

### Expression Quantitative Trait Loci Analysis

Expression quantitative trait loci (eQTL) analysis was performed to associate single nucleotide polymorphisms with individual gene expression levels. R 3.6.3 (R Core Team, 2020) and the MatrixEQTL package were used to identify eQTLs. The SNPs of 89 individuals of *P*. *tomentosa* were used as the genotype. *NRT* genes that were expressed in more than 70% of the 89 individuals were retained for eQTL analysis. The 89 individuals and their leaf nitrogen contents were used as covariates. eQTLs detected within a 250-kb window surrounding the transcription start sites of their targets were regarded as cis-eQTLs, and all others were treated as trans-eQTLs. The processing of eQTLs is described in Supplementary Method 2. The domains and possible binding motifs of candidate transcription factors were analyzed using Pfam^10^.

## Results

### Identification, Sequence Features, and Phylogenetic Analysis of *PtoNRT* Gene Family Members

To identify NRT genes in *P. tomentosa*, we performed genome-wide prediction of *PtoNRT* genes based on 62 identified *NRT* genes in *A*. *thaliana*. Using the resulting candidate *PtoNRT* genes, we performed a reciprocal BLAST search against the *Arabidopsis* genome, and precise matches to 62 *AtNRT* were obtained. In total, 98 *PtoNRT* genes were identified, of which 87 genes belong to the *NRT1*/*NPF* subfamily, seven to *NRT2*, and four to *NRT3* (Supplementary Table 2). The number of *NRT1* subfamily members expanded from 53 in *Arabidopsis* to 87 in *P. tomentosa*. The number of genes in the *NRT2* subfamily remained unchanged. The *NRT3* subfamily expanded from two to four genes. The predicted molecular weight of *PtoNRT* genes varied from 9.28 kDa (*NRT3.1*) to 120.07 kDa (*NPF5.10*). Their estimated pI values ranged from 5.27 (*NPF1.2*) to 9.78 (*NRT2.5*).

**FIGURE 1:**
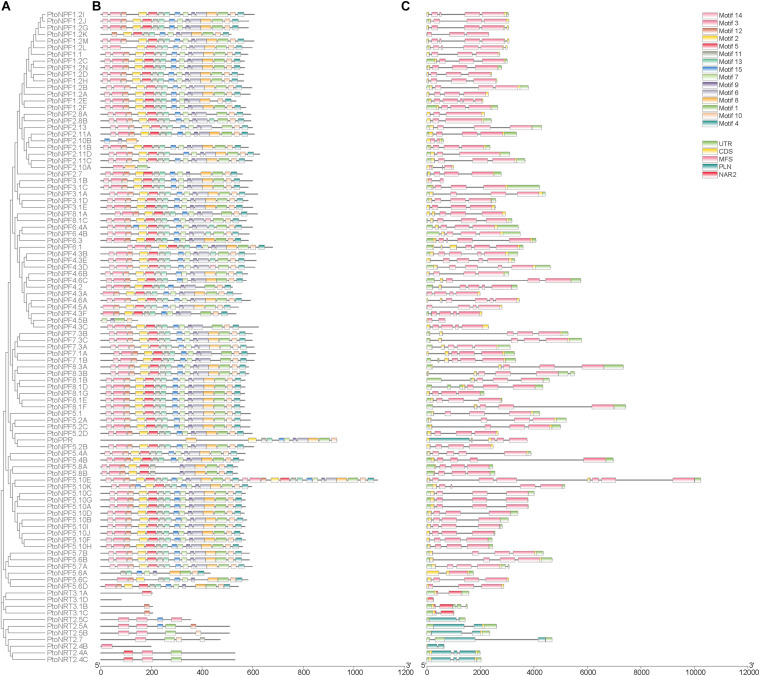
Phylogenetic, gene structure, and motif analyses of *PtoNRT* genes. **(A)** Phylogenetic relationships among PtoNRTs. The phylogenetic tree (left panel) was constructed with MEGA X software using the maximum likelihood (ML) method with 1000 bootstrap replicates. **(B)** Motif analysis of PtoNRT protein sequences. All motifs were identified using MEME software (http://meme-suite.org/). The length of each motif is shown proportionally. **(C)** Gene structure and domain analyses of *PtoNRT* genes. Gene structure maps were drawn using TBtools. A scale bar is provided at the bottom.

To investigate the evolutionary relationships among *PtoNRT* family members, an ML tree was constructed from PtoNRT protein sequences using MEGA X software. The phylogenetic tree showed that the three subgroups (NRT1/PT, NRT2 and NRT3) are distinctly separated (Figure 1A). The NRT1/PT subgroup can be further distinguished into four branches according to conserved motifs and domains. NRT3 subfamily members all contain NAR2 domains. The first plant NAR2-type member identified was as WOUND-RESPONSIVE 3 protein (WR3) (Marois et al., 2002). *NRT2* subfamily genes contain a PLN domain, while MFS is typically the main domain of *NRT1*/*NPF* subfamily genes. Most *NRT1*/*NPF* genes have 12-15 conserved motifs (Figure 1B), which are mainly transmembrane regions. In particular, PtoNPF5.10E has two identical domains and is twice as long as homologous genes. Interestingly, PtoPPR contains both PLN and MFS domain (Figure 1C) and, thus, presumably has both NRT2 and NPF functions.

**FIGURE 2:**
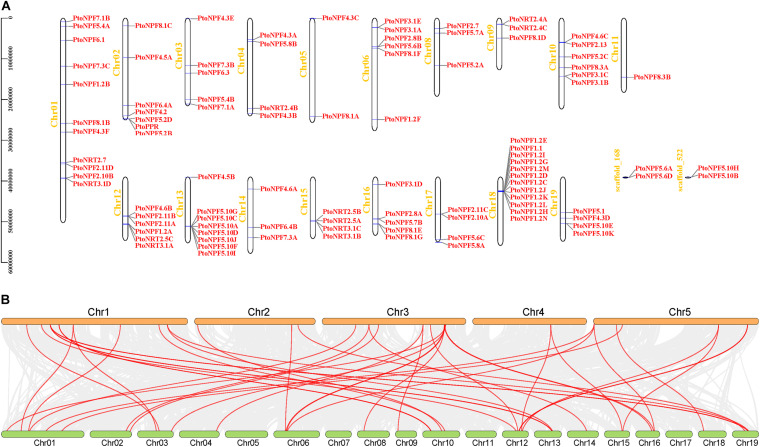
Chromosomal distribution of *PtoNRT* genes and collinearity analysis between poplar and *Arabidopsis*. **(A)** Chromosomal distribution of *PtoNRT* genes. The TBtools genome visualization tool was used to illustrate the chromosomal distribution of *PtoNRT* genes. The chromosome number is shown to the left of each chromosome. The chromosomal location of each *PtoNRT* is presented from the top to the bottom of the corresponding chromosome. The scale bar beside each chromosome indicates the length in megabases (bp). **(B)** Collinearity analysis between poplar and *Arabidopsis*. The five chromosomes of *Arabidopsis* are shown at the top, and the 19 chromosomes of *P. tomentosa* are at the bottom. Members of the *NRT* gene family are marked with red lines.

We investigated the chromosomal locations of *PtoNRT* members. The 98 *PtoNRT* genes are distributed unevenly on the nineteen chromosomes (Figure 2A). Chromosome (Chr) 18 contains the largest number of *PtoNRT* genes, with 12, followed by Chr01, with 11 genes. Chr05 contains two genes and Chr11 has only one. In addition, four genes (*PtoNPF5.6A*, *PtoNPF5.6D*, *PtoNPF5.10B*, *PtoNPF5.10H*) were located on two scaffolds. Notably, eleven *PtoNPF1.2* subgroup genes are located on chromosome 18 in adjacent positions, suggesting a replication event during evolution, and the same pattern was apparent for *PtoNPF5.10* subgroup on Chr13. We investigated further the collinearity of *PtoNRT* and *AtNRT* genes through whole genome synteny analysis. We found that many *PtNRT* genes show collinearity within the *P. tomentosa* genome (Supplementary Figure 1), suggesting that intraspecific replication events have occurred in *Populus*. A few single-copy genes remain, including *PtoNPF5.1*, *PtoNPF6.1*, and *PtoNPF6.3*. During evolution, duplication was the main impetus for *NRT* gene expansion. Under different selective pressures, homologous genes may evolve different functions, thereby increasing the diversity of gene functions. Our selection pressure analysis suggests that *PtoNPF2.8B*, *PtoNPF2.11C* and *PtoNPF1.2I* are positively selected, while all other *PtoNRT* genes are under purifying selection (Supplementary Table 3). The collinearity of orthologs between the genomes of *Arabidopsis* and *P. tomentosa* shows that only half of NRT genes are significantly conserved between the two species (Figure 2B). Two *AtNRT* genes are homologous to three *PtoNRT* genes, and nine *AtNRT* genes are duplicated in *Populus*, remaining 31 pairs of orthologs (Supplementary Table 4).

### Tissue or Organ Specific Expression of *PtoNRT* Family Members

To determine the potential functions of *NRT* genes in *Populus*, we detected the expression profiles of all *NRT* family genes in various tissues (bark, old leaf, mature xylem, expanded leaf, flower, cambium, young leaf, immature xylem, apex, phloem, root, petiole) using our transcriptome data. As shown in Supplementary Figure 2, *NRT* genes exhibited different expression patterns among the twelve tissues or organs sampled. The expression profiles of *NRT* genes in the root, mature xylem, cambium and immature xylem show similar trends. Expression patterns in the young leaf, apex and petiole grouped in one cluster. Expression in the old leaf, expanded leaf, bark and phloem samples displayed similar patterns. The female inflorescence, a reproductive tissue, did not cluster with any other tissues. Some homologous *PtoNRT* genes showed differing expression patterns in various tissues. Homologous genes generally cluster in different modules, suggesting that some homologous *NRT* genes are differentiated into multiple functions. For example, *NPF8.1C* has high expression levels in petiole, whereas *NPF8.1F* has a relatively high expression level in the mature xylem and root. *AtNPF8.1* and *AtNPF8.3* have dipeptide transport activity but no nitrate transport activity, while *AtNPF8.1* also participates in peptide uptake of roots, and *AtNPF8.3* is involved in the regulation of flowering and embryonic development in *Arabidopsis* (Komarova et al., 2008). Expression of *NPF8.3A* was detected in mature and immature xylem, and the expression levels of *NPF8.3B* were higher in roots, mature leaves, and phloem. As previously reported, *AtNPF7.3* mediates nitrate transport from root pericycle cells to the xylem (Lin et al., 2008). However, in *P. tomentosa*, only *NPF7.3B* showed high expression in the root, while *NPF7.3C* and *NPF7.3A* had high transcription levels in the phloem and mature xylem, respectively. *AtNPF1.1* and *AtNPF1.2* mediate nitrate transport via phloem, redistributing nitrate from old leaves to developing tissues (Hsu and Tsay, 2013). Only one *NPF1.1* is present in *P. tomentosa* and it is highly expressed in the petiole, as in *Arabidopsis*. Noticeably, a total of 14 *NPF1.2* members are present in *P. tomentosa*, which show six different expression modes. This diversity indicates that the function of NPF1.2 differentiated after the replication events, enriching the pathways for nitrate redistribution in *Populus*.

### Phyllotactic Expression of *PtoNRT* Gene Family Members

The distributions of nitrate in old and young leaves showed different patterns. We investigated transcript levels in annual long-shoot leaves (Long) and perennial short-branch leaves (Short). Samples were taken successively from the apex to the end of the phyllotaxis, designated Long 1-6 and Short 1-6. We found that most *NRT* genes show inverse expression patterns between Long and Short samples (Supplementary Figure 3). Root-to-shoot nitrate transport occurs through the xylem, and is driven by transpiration. As a result, old leaves obtain more nitrate from xylem than younger leaves. Among the *NPF3.1* subfamily, the expression level of *NPF3.1C* was higher in annual long shoot leaves than in perennial short-branch leaves. Thus, *NPF3.1D* is expressed in old leaves, while *NPF3.1C* is expressed in young leaves. Considering that *NPF3.1* is involved in the accumulation of nitrate and gibberellin in leaves (Pike et al., 2014; Tal et al., 2016), we speculate that NPF3.1C and NPF3.1D cooperate to transport nitrate and gibberellin from short branches to long shoots, promoting the growth of long shoots. Analogously, the expression level of *NPF5.7B* is higher in long-shoot leaves, and gradually decreases during leaf maturation. In contrast, *NPF5.7A* has high expression in short-branch leaves, which is highest in the oldest leaves. AtNPF4.6 is a constitutionally expressed low-affinity nitrate transporter (Huang et al., 1999). The expression levels of *NPF4.6B* and *NPF4.6C* in long shoot leaves are slightly higher than those in short-branch leaves. In combination with other proteins showing similar expression patterns, they deliver nitrates to long shoot leaves. AtNRT2.5 is induced by nitrogen starvation and shows a high-affinity for nitrate absorption. Most NRT2 proteins must form complexes with NAR2.1 (NRT3.1) to target the plasma membrane and maintain protein stability (Kotur et al., 2012). The expression levels of *NRT2.5A* and *NRT2.5B* increase gradually in long-shoot and short-branch leaves. The expression trends of *NRT3.1A* and *NRT3.1B* are the same as that of *NRT2.5*, indicating that NRT2 might also interact with NRT3 in *P*. *tomentosa* to assist with the loading of nitrate into old leaves. The expression patterns of *NRT* family members in long shoot and short branch leaves are significantly different, indicating that the nutrition and metabolism of the two leaf types are divergent, and that different NRT proteins might function cooperatively to reallocate nitrate.

### Circadian Rhythm and Stress Treatments Affect Expression of *PtoNRT* Family Genes

Absorption and utilization of nutrients are regulated by the circadian rhythm. Plants must also balance growth with resistance when under stress. We performed transcriptome analysis to identify expression modules within *NRT* family genes of *P*. *tomentosa* related to the circadian rhythm and stress treatments (Supplementary Figures 4, 5). The expression levels of *NRT* family genes fluctuate significantly. *NPF5.7A* is highly expressed at night, while *NPF5.7B* is highly expressed during the day. Expression of *NPF5.7A* is down-regulated with high-temperature treatment, which may reduce the outflow of nitrate from leaves to help maintain a steady state. Under high-salt stress, three genes of the *NRT3.1* subfamily cluster into the same module. Their expression levels initially increased with treatment time, peaked at 3 h, and then decreased slowly. Thus, NRT3.1 can respond to high-salt stress and is a potential regulator of osmotic pressure that helps maintain homeostasis in plants.

In *P*. *tomentosa*, the expression levels of *NPF2.10A*/*B* are generally low. *NPF2.11A*/*B* are up-regulated at night, while *NPF2.11C* and *NPF2.11D* are up-regulated in the morning and at dusk, respectively. Another substrate of AtNPF4.6 is abscisic acid (ABA) (Kanno et al., 2013). In our data, the expression patterns of *NPF4.6B*/*C* are similar, decreasing with ABA treatment time. Both genes are up-regulated at night and peak at the end of the dark period within 2 h of light, and then sharply decrease. *NPF2.11A* and *NPF6.3* gene expression patterns were similar in ABA, heavy-metal stress and circadian rhythm experiments, suggesting that they may be regulated by the same signaling factor and perform similar functions. In *P*. *tomentosa*, *NRT* genes in leaves are regulated by the circadian rhythm, which may be conducive to nutrient distribution. They work together to respond to stress, balancing nutrition and resistance to promote plant growth.

**FIGURE 3:**
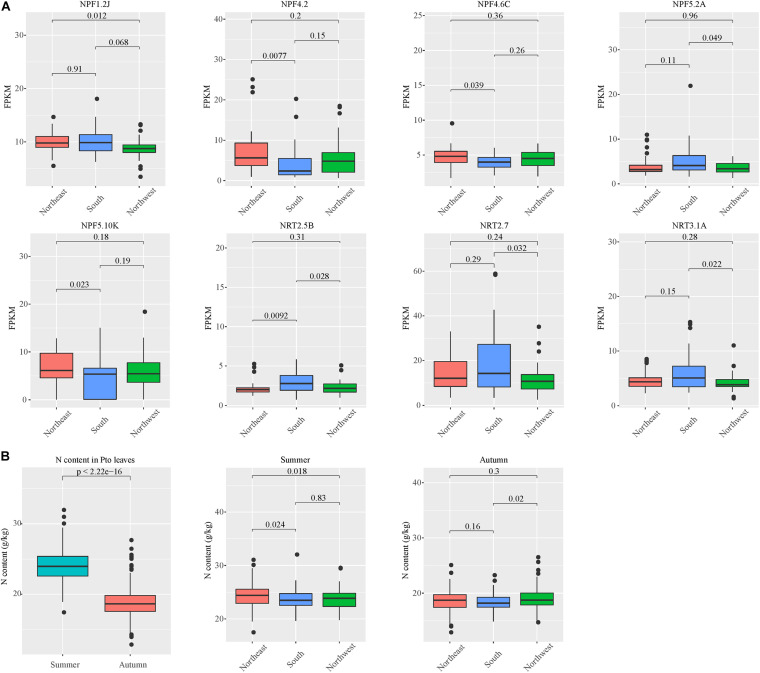
Population expression patterns of *NRT* genes and leaf nitrogen content among climatic zones and seasons. **(A)** Individual plants were divided into three main climate zones, namely the northeast, northwest and south. The *ANOVA* method in R software was used to identify significant differences in gene expression levels. The abscissa is the climate zone, the ordinate is FPKM, and the significance level is denoted above. **(B)** Leaf nitrogen content between climatic zones and seasons. The abscissa is the season or climate zone, the ordinate is N content (g/kg), and the significance level is denoted above each pair of zones.

In our analyses of tissue specificity, phyllotaxis, circadian rhythm and stress treatment transcriptome data, six genes were never expressed (*PtoNPF2.10B*, *PtoNPF1.2A*, *PtoNPF4.5B*, *PtoNPF3.1B*, *PtoNRT3.1D*, *PtoNPF4.3F*) (Supplementary Figure 6). Furthermore, the expression patterns of the *NRT* gene family in the *P. tomentosa* population are influenced by geographical and climatic factors in the distribution area. Among three climatic zones, the *NPF1.2J*, *NPF4.2*, *NPF4.6C*, *NPF5.2A*, *NPF5.10K*, *NRT2.5B*, *NRT2.7* and *NRT3.1A* genes show significant differences (Figure 3A). This finding indicates that these *NRT* genes have undergone adaptive selection. Temperature, precipitation, circadian rhythm and other factors have caused irreversible changes to the expression levels of these genes.

### Genetic Basis for Natural Variants of Leaf Nitrogen Content Traits in *P. tomentosa*

The leaf nitrogen content of *P. tomentosa* varies significantly among individuals in different climatic regions (Figure 3B). In autumn, significant differences in leaf nitrogen content can be observed between individuals from the southern and northwestern climate zones. In summer, the northeast region shows significant differences in relation to the south and northwest, while differences between the south and northwest are not apparent. In addition, leaf nitrogen content in summer was significantly higher than that in autumn (Figure 3B). These results indicate that nitrogen redistribution occurred during autumn leaf senescence and was affected by climate and region.

**FIGURE 4:**
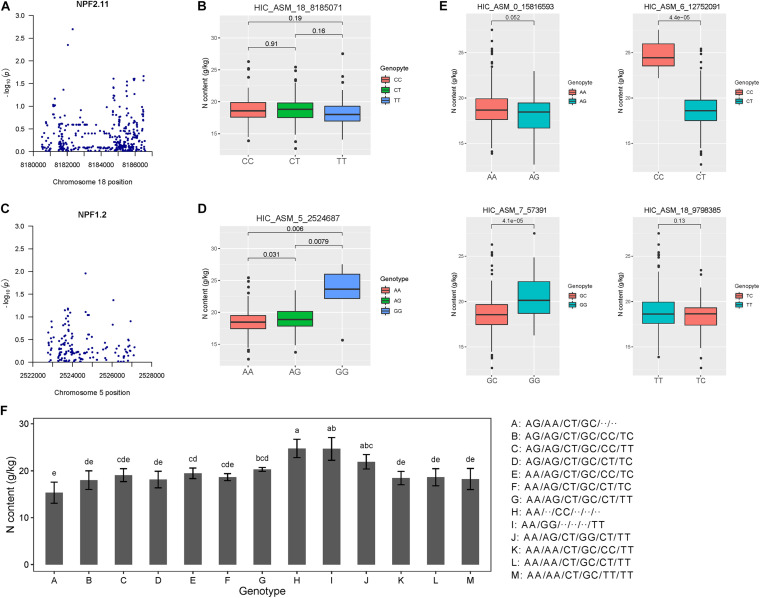
Association analysis and epistatic detection revealed the genotypic effects of individual SNPs on leaf nitrogen content. **(A)** Manhattan plots for *NPF2.11*. **(B)** Box plots showing the effects of a selected epistatic SNP of *NPF2.11* on leaf nitrogen content. **(C)** Manhattan plots for *NPF1.2*. **(D)** Box plots of one epistatic effect SNP in *NPF1.2* for leaf nitrogen content. **(E)** Genotypic effects of each causal SNP on autumn leaf nitrogen content. **(F)** Combined genotypic effects on autumn leaf nitrogen content in the association population of *P. tomentosa*. Six allelic variations were ordered according to the results shown in Panel **(B,D,E)**. We used the following SNPs: HIC_ASM_0_15816593, HIC_ASM_5_2524687, HIC_ASM_6_12752091, HIC_ASM_7_57391, HIC_ASM_18_8185071 and HIC_ASM_18_9798385. The genotype “..” indicates that neither genotype has an effect on leaf nitrogen content.

We used association genetics (additive, dominant and epistatic effects) to analyze the genetic effects of leaf nitrogen content traits in the association population of *P. tomentosa*. Based on genome resequencing data (coverage > 15×) of 285 unrelated individuals, we selected 20,441 high-quality SNPs from 98 NRT family genes (sub-allele frequency MAF > 5%, missing data < 10%) for further analysis (Supplementary Datasheet 2). We found nine significant loci distributed across five NRT genes for the summer trait, and 16 loci corresponding to eight NRT genes for the autumn trait (Table 1). Each association explained phenotypic variations of 4.33%-6.76% (based on *R*^2^), with an average of 5.48%. Among significant loci for autumn, five loci were located on the *NPF6.4A* gene. These associated loci exhibited various effects on traits (Figures 4A-E). Among the 25 locus–trait associations, 11 associations (44%) had joint additive and dominant effects (Table 1), they were distributed within six *NRT* genes, with four occurring in summer and two in autumn. Thus, members of the *NRT* family function differently in different seasons.

**TABLE 1:** Nitrate transporter family gene association analysis results.

**Trait**	**Gene**	**Locus**	**Site**	**p**	**Marker R^2^**	**Additive effect**	**Dominant effect**
Summer	NPF5.7B	HIC_ASM_4	3888128	4.48E-5	0.06051		
	NPF8.3A	HIC_ASM_3	10772965	9.19E-5	0.06765	–2.561	0.751
	NPF8.3A	HIC_ASM_3	10770957	1.53E-4	0.06383	–0.715	–1.697
	NPF2.11B	HIC_ASM_18	8185059	1.53E-4	0.06750	1.496	1.077
	NRT2.5C	HIC_ASM_18	9835285	2.21E-4	0.06108	–2.136	0.998
	NPF2.11B	HIC_ASM_18	8185066	4.59E-4	0.06275	–1.480	1.091
	NPF2.11B	HIC_ASM_18	8185043	6.79E-4	0.04331		
	NPF2.11B	HIC_ASM_18	8185071	9.09E-4	0.05211	0.456	0.069
	NRT3.1B	HIC_ASM_13	10297158	9.98E-4	0.04986	2.346	0.969
Autumn	NPF5.1	HIC_ASM_0	15816593	1.47E-4	0.06080		
	NPF6.4A	HIC_ASM_15	19847470	4.86E-4	0.05207		
	NPF7.3C	HIC_ASM_6	12752091	5.19E-4	0.05063		
	NPF6.4A	HIC_ASM_15	19847476	5.98E-4	0.05201		
	NPF5.4B	HIC_ASM_9	1866319	6.16E-4	0.04926		
	NPF6.4A	HIC_ASM_15	19847459	6.38E-4	0.05013		
	NPF1.2A	HIC_ASM_18	9798385	6.57E-4	0.04874		
	NPF4.5B	HIC_ASM_7	57391	7.64E-4	0.04754		
	NPF1.2F	HIC_ASM_5	2524687	7.77E-4	0.06109	0.087	–0.110
	NPF6.4A	HIC_ASM_15	19847450	7.97E-4	0.04976		
	NPF5.1	HIC_ASM_0	15813525	9.21E-4	0.04709		
	NPF5.1	HIC_ASM_0	15813529	9.21E-4	0.04709		
	NPF6.4B	HIC_ASM_15	21476767	9.65E-4	0.05942	0.326	0.318
	NPF6.4B	HIC_ASM_15	21476768	9.65E-4	0.05942	–0.326	0.318
	NPF6.4B	HIC_ASM_15	21476770	9.65E-4	0.05942	–0.326	0.318
	NPF6.4A	HIC_ASM_15	19847455	9.94E-4	0.04690		

We tested the pairwise epistasis between the variants of each SNP for each trait. Among 20,441 SNPs in *NRT* genes, 1500 significant pairwise epistatic combinations were detected for the summer and autumn leaf nitrogen content traits at *P* < 1.0 × 10^–4^ (Supplementary Table 5). Among these 1500 epistatic interactions, 428 additive × additive (AA), 415 additive × dominance (AD), 498 dominance × additive (DA) and 159 dominance × dominance (DD) interaction effects were identified for the two traits. Notably, autumn leaf nitrogen content trait accounted for 93.57% of DA effects. Among the 25 locus–trait associations, two (18_8185071_T and 5_2524687_G) showed epistatic effects. The GG genotype of 5_2524687_G contributed significantly to leaf nitrogen content (Figure 4D). We selected four independent SNPs among the significant loci for the autumn leaf nitrogen content trait, with different effects on phenotype (Figure 4E). We identified possible genotype combinations of the six significant SNPs for the autumn trait (Figure 4F). The genotype alternations of two major loci (HIC_ASM_5_2524687, HIC_ASM_6_12752091) showed especially strong effects on phenotype. Evidently, AG/AA/CT combinations at the first three major loci contribute to lower leaf nitrogen content, while AA/⋅⋅/CC and AA/GG/⋅⋅ combinations result in higher leaf nitrogen content. These findings support the possibility that genotype combinations of single locus allelic variations are the main factors driving autumn leaf nitrogen content. These results indicate that *NRT* family genes have functions in leaf nitrogen content.

### Expression Variation of WGCNA Modules Within a Population

**FIGURE 5:**
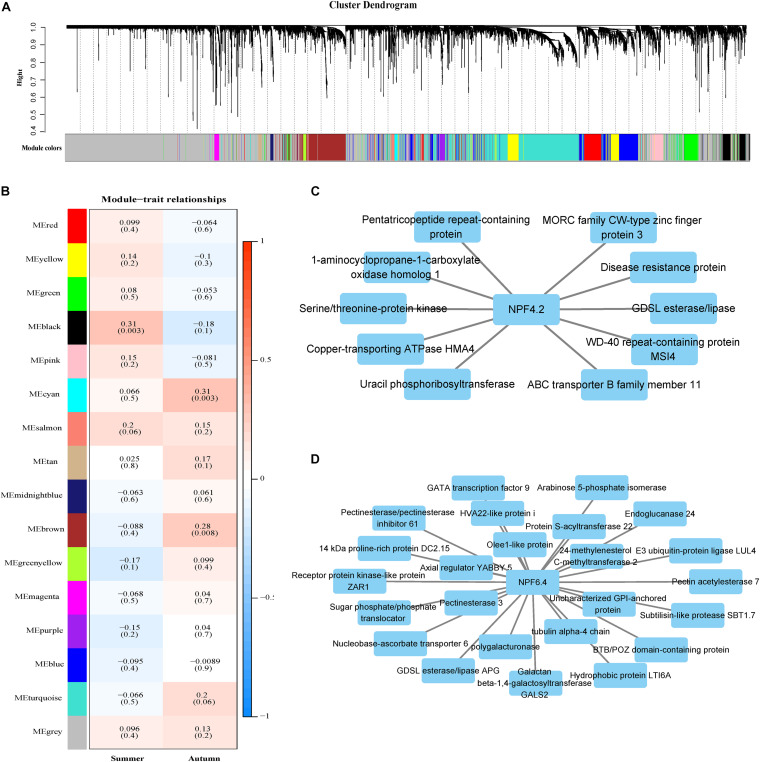
Gene modules identified through weighted gene co-expression network analysis (WGCNA). **(A)** Gene dendrogram obtained through dissimilarity clustering based on consensus topological overlap, with the corresponding module colors indicated by the row color. Each colored row represents a color-coded module which contains a group of highly connected genes. A total of 25 modules were identified. **(B)** Relationships among modules and leaf nitrogen content during two seasons. Each row in the table corresponds to a module, and each column to a season. The module name is shown on the left side of each cell. Numbers in the table represent the correlations between the corresponding module eigengenes and stage, with *p* values listed below the correlations in parentheses. The table is color-coded to represent correlation strength according to the color legend. The intensity and direction of each correlation are indicated on the right side of the heatmap (red, positively correlated; blue, negatively correlated). **(C)** Genes with strong correlations (*p* < 10E-05) with the *NPF4.2* gene in the black module. **(D)** Genes with strong correlations (*p* < 10E-30) with the *NPF6.4* gene in the brown module.

Weighted gene co-expression network analysis was performed using transcriptome data from the *P. tomentosa* population. A network was constructed from the filtered expression data. We selected a soft threshold power 12 to define the adjacency matrix based on the criterion of approximate scale-free topology (Supplementary Figure 7), with a minimum module size of 30, and module detection sensitivity of *deepSplit* 4. After merging, 16 modules were identified (Figure 5A). The connectivity of eigengenes was analyzed to identify interactions among these 16 co-expressed modules. A significant difference among the 16 modules was found (Figure 5B). Three modules were significantly correlated with phenotypes. The black modules were positively related to the summer season, while the cyan and brown model was positively related to the autumn season. A total of 166 genes were annotated in the black module, 459 in the brown module and 30 in the cyan module (Supplementary Table 6). Among those genes, the black module contained *NPF4.2* (Figure 5C) and the brown module contained *NPF6.4* (Figure 5D). We performed Gene Ontology (GO) annotation of the genes in the black and brown modules. The black module was mainly enriched in oxidation-reduction and cellular amide metabolic process genes, while the brown module was mainly enriched in cell wall organization or biogenesis and cellular polysaccharide metabolic process genes (Supplementary Figure 8). In the black module, some genes associated with metabolism and energy transfer were significantly associated with *NPF4.2*, such as a serine/threonine-protein kinase gene. *NPF6.4*, in the brown module, was related to genes in the sugar synthesis pathway and several transcription factors, including axial regulator YABBY5 and GATA transcription factor 9. These findings indicate that *NRT* family genes are involved in the maintenance of the cell wall and cellular homeostasis.

### Expression Quantitative Trait Nucleotide (eQTN) Mapping

To investigate the causative genetic variants underlying the transcription levels of NRT genes, eQTL analysis was performed between whole-genome SNPs and the expression levels of 54 NRT genes (expressed in ≥ 70% of the 89 individuals). The leaf nitrogen contents in summer and autumn were regarded as the phenotype. We found that four main NRT genes defined thousands of eQTL signals (Supplementary Datasheet 3). We selected SNP sites with higher P values for annotation (Supplementary Table 7). Among these sites, two were significantly related to *NPF2.11* and were annotated as *NAC domain-containing protein 86* and transcription factor *bHLH162*, respectively. Both types of transcription factors are involved in the regulation of the NUE process (Samira et al., 2018; Tang et al., 2019). The transcription factor gene *MYB17* showed strong associations with *NPF6.1* in both summer and autumn. This result indicates a potential upstream regulatory network of *NRT* family genes. A WRKY transcription factor was associated with the NRT1.1 gene. This gene had high expression in roots. We extracted the sequence of this gene and found that it has high homology with a WRKY21-related gene in *P. trichocarpa*, indicating that WRKY21 may be involved in the regulation of *NRT1.1* at the transcription level. In addition, we compared the annotated genes with those in the WGCNA modules and found that they overlapped with one gene in the brown module (evm.model.HIC_ASM_15.1752). The eQTL signal of this gene was mapped to *NPF6.1* which encodes a lipid raft-regulatory protein, remorin (REM). Our results suggested that the activities of NPF6.1 and NPF6.4 might be related to membrane lipid nanodomain-localized REM protein in cell-to-cell signaling.

### Poplar-Specific Potential Regulatory Factors of *NRT* Genes

In the results from WGCNA and eQTL, the significantly related genes included more than 20 transcription factor and response protein genes (Supplementary Table 8). We analyzed the domains and possible binding motifs of these transcription factors using Pfam. Myb-like DNA-binding domains, helix-loop-helix DNA-binding domains and GATA zinc finger domains were significantly enriched. Members of the bHLH family bind to the sequence “CANNTG,” also known as the E-box motif (Chaudhary and Skinner, 1999). GATA-type zinc finger (Znf) transcription factor specifically binds to the DNA sequence (A/T) GATA (A/G) (Yamamoto et al., 1990).

**FIGURE 6:**
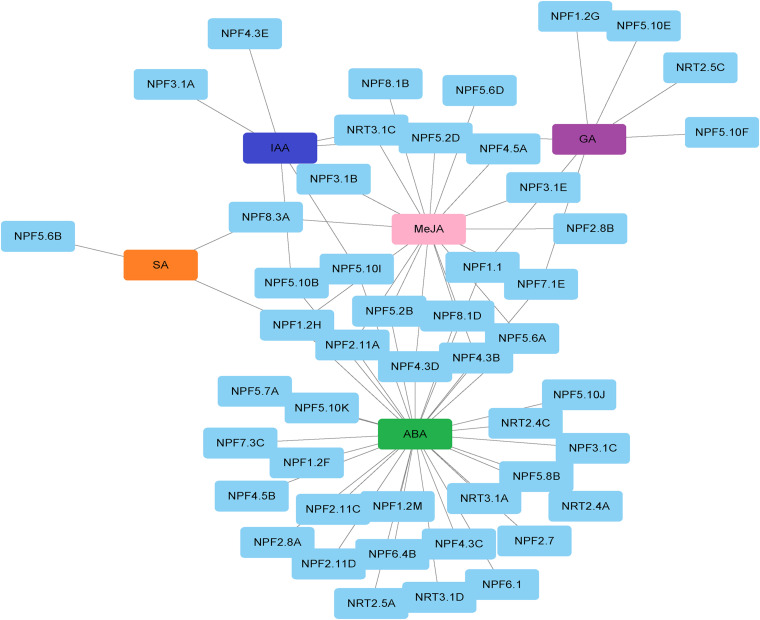
Regulation network of poplar *NRT* genes and plant hormones. IAA, auxin; MeJA, methyl jasmonate; GA, gibberellin; ABA, abscisic acid; SA, salicylic acid.

To investigate upstream regulatory factors of *NRT* genes, we predicted *cis*-regulatory elements (CREs) in the promoter sequence of *NRT* family genes in *P. tomentosa* using plantCARE (Supplementary Table 9). We screened the resulting response elements (Supplementary Figure 9). Among them, the number of light-responsive elements was largest, accounting for more than half of all response elements. This finding is in accordance with the large changes observed in the expression levels of *NRT* family genes between day and night (Supplementary Figure 4). More than half of the remaining elements are hormone-responsive elements, including those responding to auxin, methyl jasmonate (MeJA), gibberellin (GA), ABA and salicylic acid (Figure 6). Aside from salicylic acid, the other four plant hormones are transport substrates of some NRT proteins. The promoters of *PtoNRT3.1A*, *PtoNPF1.2F*, and *PtoNPF5.10I* are enriched among ABA responsive elements. The expression levels of these three genes decrease significantly with increasing ABA treatment time (Supplementary Figure 5), suggesting that they are target genes in the ABA signaling pathway. Many *NRT* gene promoters are enriched in response elements to more than one plant hormone. The *PtoNPF5.6A* promoter is enriched in ABA- and MeJA-responsive elements at the same location, suggesting that ABA and MeJA antagonistically regulate the expression of *PtoNPF5.6A*. The *PtoNPF5.10I* promoter was enriched in ABA- and auxin-responsive elements, the *PtoNRT3.1C* promoter in MeJA- and auxin-responsive elements, and the *PtoNPF8.3A* promoter in MeJA- and SA-responsive elements. The *PtoNPF4.3B*, *PtoNPF4.3D*, *PtoNPF8.1D* and *PtoNPF2.11A* promoters were enriched in ABA- and GA-responsive elements. The *PtoNPF7.1B* promoter was enriched in ABA-, GA- and MeJA-responsive elements. Thus, *NRT* genes are coordinated by a variety of plant hormones.

## Discussion

### The *NRT* Gene Family Has Expanded in Poplar

The *NRT* gene family has many members with distinct functions. At present, 62 NRT genes have been identified in *Arabidopsis* (AtGenIE). We found 98 *NRT* genes in *P. tomentosa* that are homologous to those in *Arabidopsis* through a homology comparison. Compared with previous research (Bai et al., 2013) and existing annotations in the database (Phytozome), the number of *NRT* genes we identified represent only a slight increase or decrease (Supplementary Table 4).

Some single-copy genes have shown few sequence changes during evolution. We assume that the functions of these genes are relatively well conserved between poplar and *Arabidopsis*. For example, *PtoNPF6.3* is the closest ortholog of *AtNPF6.3* in poplar, and both encode dual-affinity nitrate transporters. The expression pattern of *PtoNPF6.3* is also similar to that in *Arabidopsis* (Guo et al., 2001; Krouk et al., 2010). Therefore, PtoNPF6.3 is likely to be a key transceptor in the nitrate signaling pathway. AtNPF4.1 was identified as having ABA and GA transport activity through yeast screening experiments (Kanno et al., 2012). The homologous gene of *AtNPF4.1* in *P. tomentosa*, *PtoNPF4.2*, is highly expressed during daytime and in old leaves. PtoNPF4.2 may also be a transporter of ABA and GA that helps maintain the physiological balance of old leaves. Notably, the expression level of *PtoNPF4.2* is extremely different in the northeastern and southern climate regions, which may be due to differences in the leaf growth cycle caused by climatic factors. This finding indicates that the genes of perennial woody plants have developed functions similar to those of annual herbaceous plants during their evolution. The functions of NRT2.7 seem to have diversified during evolution. *AtNRT2.7* is specifically expressed in seeds and is responsible for loading nitrate into the vacuoles of seed cells (Chopin et al., 2007). However, in *P. tomentosa*, *NRT2.7* expression is up-regulated at night and is elevated in growing leaves. Thus, NRT2.7 participates in nutrient transport during leaf growth and development in poplars. Furthermore, the expression level of *NRT2.7* differs significantly between individuals in the southern and northwestern climate zones. This difference may be related to differing leaf growth patterns caused by climatic and geographical factors.

The *PtoNPF2.11B* gene is orthologous to *AtNPF2.11*, *AtNPF2.10* and *AtNPF2.9* (Supplementary Table 4). *AtNPF2.10* and *AtNPF2.11* show low-affinity nitrate transport and glucosinolate (GLS) transport activities (Nour-Eldin et al., 2012). AtNPF2.10 also participates in the transport of GAs and jasmonic acid-isoleucine (JA-Ile), mediating the transfer of JA/JA-Ile from damaged leaves to undamaged leaves in response to wound signals (Ishimaru et al., 2017; Saito et al., 2015). AtNPF2.11, AtNPF2.10, AtNPF2.9 all have NO_3_ - and GLS transport functions (Nour-Eldin et al., 2012). AtNPF2.9 participates in phloem loading of nitrate in the root, while AtNPF2.10 and AtNPF2.11 are involved in GLS translocation to seeds. The *PtoNPF2.11B* gene is expressed in the bark, female flowers, apex and petiole, suggesting that it may have evolved a novel function. On the other hand, some subfamily members (*PtoNPF5.10*, *PtoNPF1.2*) located in the same chromosome region do not have orthologous genes (Figure 2A). This finding shows that tandem duplication events within the poplar genome have led to the expansion of these subfamily members. Although the number of genes in the *NRT2* subfamily in poplar has not changed, only three members have orthologous relationships with *NRT2* genes of *Arabidopsis* (*AtNRT2.6*: *PtoNRT2.4A*, *PtoNRT2.4B*; *AtNRT2.7*: *PtoNRT2.7*). Other members may have undergone major mutations or translocations during evolution. *PtoNRT3.1A* and *PtoNRT3.1C* are orthologous to both *AtNRT3.1* (AT5G50200) and the AT4G24730 gene. However, AT4G24730 has no detailed annotation information for *Arabidopsis*. Therefore, we can basically confirm that the AT4G24730 gene belongs to the *NRT3* subfamily.

### Diverse Expression Patterns of *NRT* Family Genes in Poplar

Bark is a unique organ of woody plants. An important feature of the seasonal nitrogen cycle of poplars is the accumulation of bark storage protein (BSP) (Babst and Coleman, 2018). Much of the nitrogen that moves to the stem from senescent leaves in autumn is used for synthesis of BSP. *PtoNPF1.2B* is highly expressed in the bark (Supplementary Figure 2), and one of its orthologous genes in Arabidopsis is *AtNPF1.2*. *AtNPF1.2* is expressed in the companion cells of the major veins of expanded leaves, and is involved in transferring nitrate that has accumulated in mature and expanded leaves to the phloem of major veins, allowing nitrate redistribution from larger expanded leaves to the youngest tissues (Hsu and Tsay, 2013). We speculated that PtoNPF1.2B is responsible for nitrate loading in bark. Surprisingly, *PtoNPF1.2B* is highly expressed in all tissues except old leaves and mature xylem. To adapt to the high demand for nitrate transport in perennial plants, *NPF1.2* is expressed in more locations in poplars than *Arabidopsis*. The tissue-specific expression and subcellular localization of NPF1.2 protein in poplars is an interesting topic for future research. Presumably, NPF1.2 is a high-capacity channel for nitrate transportation in poplar.

Long-shoot leaves and short branch leaves are unique tissue classifications of trees. Long-shoot leaves are leaves that grow from current-year shoots, whereas short-branch leaves are leaves that grow from perennial branches. The leaves on long shoots are more tender and faster growing. *NRT* genes, which are highly expressed in long-shoot leaves, may be responsible for unloading of nitrate into developing leaves. The nitrate flow rate will be greater in the more mature short-branch leaves. We found that *PtoNPF6.3*, *PtoNRT3.1A* and *PtoNRT2.5A*/*B* all have higher expression levels in short-branch leaves relative to long-shoot leaves (Supplementary Figure 3). Previous studies have shown that expression of *AtNRT3.1* (ortholog of *PtoNRT3.1A*) is essential for the correct orientation of AtNRT2.1 in the plasma membrane and maintenance of AtNRT2.1 stability (Yong et al., 2010). The AtNRT2.1/AtNAR2.1 complex is a tetramer composed of two subunits each of AtNRT2.1 and AtNAR2.1, which functions in high-affinity nitrate influx. Moreover, except for AtNRT2.7, all other NRT2 transporters interact with AtNRT3.1 (Kotur et al., 2012). As NPF6.3 is a dual-affinity transporter, it is converted into a low-affinity protein when the nitrate concentration is high. At that point, NPF6.3 transmits a nitrate signal to the nucleus, which may induce the expression of *NRT3.1* and promote formation of NRT2/NRT3.1 high nitrate affinity complexes to supplement the function of NPF6.3. When the nitrate concentration is low, efficient nitrate transport channels are not needed. The low-nitrate signal transmitted by NPF6.3 may inhibit the expression of *NRT3.1* and reduce the number NRT2/NRT3.1 complexes. Simultaneous conversion of NPF6.3 into a high-affinity protein occurs to maintain the transport flux of nitrate. Furthermore, the expression of *PtoNRT3.1A* was significantly lower in the drier and hotter northwest climate region than in the humid and rainy southern climate region. Moreover, under the influence of five stressors, namely ABA, drought, heavy metal, high salt and high temperature, the expression of *PtoNRT3.1A* showed a downward trend, suggesting that environmental factors such as drought and high temperature reduce the mobility of nitrate.

### Genetic Effects and *Trans*-Acting Factors of *NRT* Genes

Trees reabsorb specific nutrients from the leaves during seasonal leaf senescence (Wang et al., 2018). Compared with other organs, leaves have a higher nitrogen content. Trees can transfer up to 50-80% of nitrogen in the leaves to the stem for storage during seasonal dormancy, and then use it for growth in spring, greatly improving their nitrogen use efficiency (NUE). A previously reported allele variation of *OsNRT1.1B* significantly improved grain yield and NUE (Hu et al., 2015). Genotypic variations underlying phenotype diversification can be used for molecular marker assisted breeding. Thus, our primary goal was to elucidate the role of *PtoNRT* genes in leaf nitrate reflux.

Association analysis is a reliable strategy for identifying causal genes in studies of tree population genetics (Ingvarsson and Street, 2011). In our association results, the leaf nitrogen contents of individuals with different haplotypes at three SNP loci (HIC_ASM_5_2524687-*NPF1.2F*, HIC_ASM_6_12752091-*NPF7.3C* and HIC_ASM_7_57391-*NPF4.5B*) differed significantly (Figures 4D,E). The GG, CC, and GG genotypes of those loci, respectively, lead to high-nitrogen phenotypes. A high-nitrogen content in leaves during autumn is not a desired result. Avoiding the combination of these genotypes can effectively reduce leaf nitrogen content in autumn (Figure 4F). *NPF1.2F* is sensitive to ABA (Supplementary Figure 5), which is the key hormone that induces leaf senescence. *AtNPF4.5* has been identified as an ABA transporter (Kanno et al., 2012). However, the role of *NRT* gene regulation by ABA in leaf senescence has not been studied. *NPF7.3C* is highly expressed in the phloem. *Arabidopsis NPF7.3* is associated with stress tolerance (Zhang et al., 2014), and the *npf7.3* mutant exhibits leaf senescence-related phenotypes (Zheng et al., 2016). The cooperative network of *NRT* genes and their regulatory factors during leaf senescence is an interesting topic that warrants further investigation.

We obtained 27 transcription factors from the results of WGCNA and eQTL. We also predicted the CREs of the promoter sequences of *NRT* family genes. A dozen *NRT* gene promoters were found to contain circadian control elements (Supplementary Table 9). This result indicates that *NRT* gene expression is indeed regulated by the circadian rhythm, with distinct patterns during the day and night that help maintain the physiological balance and promote the development of plants. Many promoters of *NRT* genes are also enriched MYB binding sites (Supplementary Figure 9), suggesting that they are directly regulated by MYB transcription factors. MYB transcription factors play key roles in various developmental processes in plants. In addition, an MYB-like transcription factor responds to low-nitrate conditions by binding to the *AtNRT1.1* gene promoter (Lee et al., 2020). The *PtoNPF7.3C* and *PtoNPF2.7* promoters were associated with the drought-inducible MYB binding site. Their expression levels first increased and then decreased with drought-stress treatment time, suggesting that these two genes respond to drought stress. The *PtoNPF5.2A* and *PtoNPF4.6B* promoters were related to the light-responsive MYB binding site. Their expression levels fluctuated with the cycle of day and night, suggesting that MYB is involved in regulating the circadian rhythm of *NRT* genes. The *PtoNPF1.2H* and *PtoNPF1.2N* promoters enriched the MYBHv1 binding site, which contains a CCAAT-box sequence. The CCAAT box is a common cis-acting element to which diverse transcription factor proteins are known to bind (Laloum et al., 2013), including nuclear transcription factor Y (NF-Y) subunit A-1 in the black module. GATA transcription factor 9 in the brown module and GATA transcription factor 16 in the black module may also regulate *NRT* genes with GATA motif-enriched promoters. The promoters of *PtoNPF5.10J* and *PtoNPF5.1* each contain three GATA-motifs. The functions and regulatory relationships of many *NRT* genes require further exploration, especially those of poplar *NRT* genes that are differentiated from genes in *Arabidopsis*.

Our research has found some interesting results of the *NRT* family in *P*. *tomentosa*, which may have potential breeding value. Association analysis and eQTL associated sites are significantly related to *NPF2.11B*. *NPF6.4* was significantly correlated with leaf nitrogen content phenotype in association analysis and WGCNA. It shows that *NPF2.11* and *NPF6.4* may play the key role in the nitrogen use efficiency of poplars. The promoter of *NRT3.1* enriches the response elements of ABA and other plant hormones. *NRT3.1* responds to high salt stress and has the same expression trend as *NRT2.5*. The expression patterns of *NRT3.1* in poplars are diverse, which may be a key factor in the regulation of nitrate absorption and distribution in poplars.

## Data Availability Statement

The original contributions presented in the study are publicly available. The CDS and protein sequences of PtoNRT genes were uploaded to GenBank Banklt (Accession Numbers: MW544773 – MW544870) (https://www.ncbi.nlm.nih.gov/genbank/). The transcriptome expression data are available in the National Center for Biotechnology Information SRA database, accession numbers PRJNA521819, PRJNA521855, PRJNA522886, PRJNA522891, PRJNA357670, SRP141094, SRP073689, and SRP060593.

## Author Contributions

DZ conceived and designed the study. LZ, PL, and PC performed the experiments. LZ and PC carried out the data analysis. LZ prepared the manuscript. YS and DZ performed a critical review of intellectual content. All authors have read, edited, and approved the current version of the manuscript.

## Conflict of Interest

The authors declare that the research was conducted in the absence of any commercial or financial relationships that could be construed as a potential conflict of interest.

## Footnotes

1 http://atgenie.org/

2 https://blast.ncbi.nlm.nih.gov/Blast.cgi

3 https://www.ncbi.nlm.nih.gov/Structure/bwrpsb/bwrpsb.cgi

4 http://bioinformatics.psb.ugent.be/webtools/plantcare/html/

5 https://www.ncbi.nlm.nih.gov/genbank/

6 http://web.expasy.org/compute_pi/

7 https://www.megasoftware.net

8 http://tree.bio.ed.ac.uk/software/figtree/

9 http://meme-suite.org/tools/meme

10 https://pfam.xfam.org/

## Supplementary Material

Supplementary Figure 1 Collinearity analysis of genes within poplar.

Supplementary Figure 2 Expression profiles of NRT family genes in *P*. *tomentosa*. Heatmap of tissue-specific expression.

Supplementary Figure 3 Expression profiles of NRT family genes in *P*. *tomentosa*. Long-shoot leaves and short branch leaves. Long, annual long-shoot leaves; Short, perennial short-branch leaves. The development of leaves, showing gradual maturation from 1 to 6.

Supplementary Figure 4 Expression profiles of NRT family genes in *P*. *tomentosa*. Heatmap of circadian rhythm-related expression. Samples were collected every 2 h, under a cycle of 14 h light and 10 h dark.

Supplementary Figure 5 Expression profiles of NRT family genes in *P*. *tomentosa*. Heatmap of stress-induced expression. ABA, ABA treatment; D, Drought; HM, Heavy metal; HS, High salt; HT, High temperature. Labels 1-5 correspond to samples collected at 1, 3, 6, 12, and 18 h, respectively.

Supplementary Figure 6 Venn diagram of non-expressed genes.

Supplementary Figure 7 Network topologies for various soft-thresholding powers.

Supplementary Figure 8 GO clustering of black and brown module genes.

Supplementary Figure 9 *Cis*-regulatory elements (CREs) of the promoters of *NRT* family genes in *P. tomentosa*.

Supplementary Table 1 Leaf nitrogen content of *P. tomentosa.*

Supplementary Table 2 Identified *NRT* family genes in *P. tomentosa.*

Supplementary Table 3 Genes in the *NRT* family of *P. tomentosa* under positive selection.

Supplementary Table 4 Collinearity of *NRT* genes between *P. tomentosa* and *Arabidopsis*.

Supplementary Table 5 Pairwise epistatic combinations of *NRT* genes.

Supplementary Table 6 WGCNA module genes.

Supplementary Table 7 Significant SNPs from the eQTL results.

Supplementary Table 8 Significantly related transcription factors from WGCNA and eQTL.

Supplementary Table 9 *Cis*-regulatory elements (CREs) of *NRT* family gene promoters.
