# Supplementary material for: Genetic Effects and Expression Patterns of the Nitrate Transporter (NRT) Gene Family in Populus tomentosa
Source: Front Plant Sci. 2021 May 13;12:661635. doi: 10.3389/fpls.2021.661635 (PMC8155728; doi:10.3389/fpls.2021.661635)
Supplement: Supplementary Figure 1 — Collinearity analysis of genes within poplar. [file Data_Sheet_1.doc]

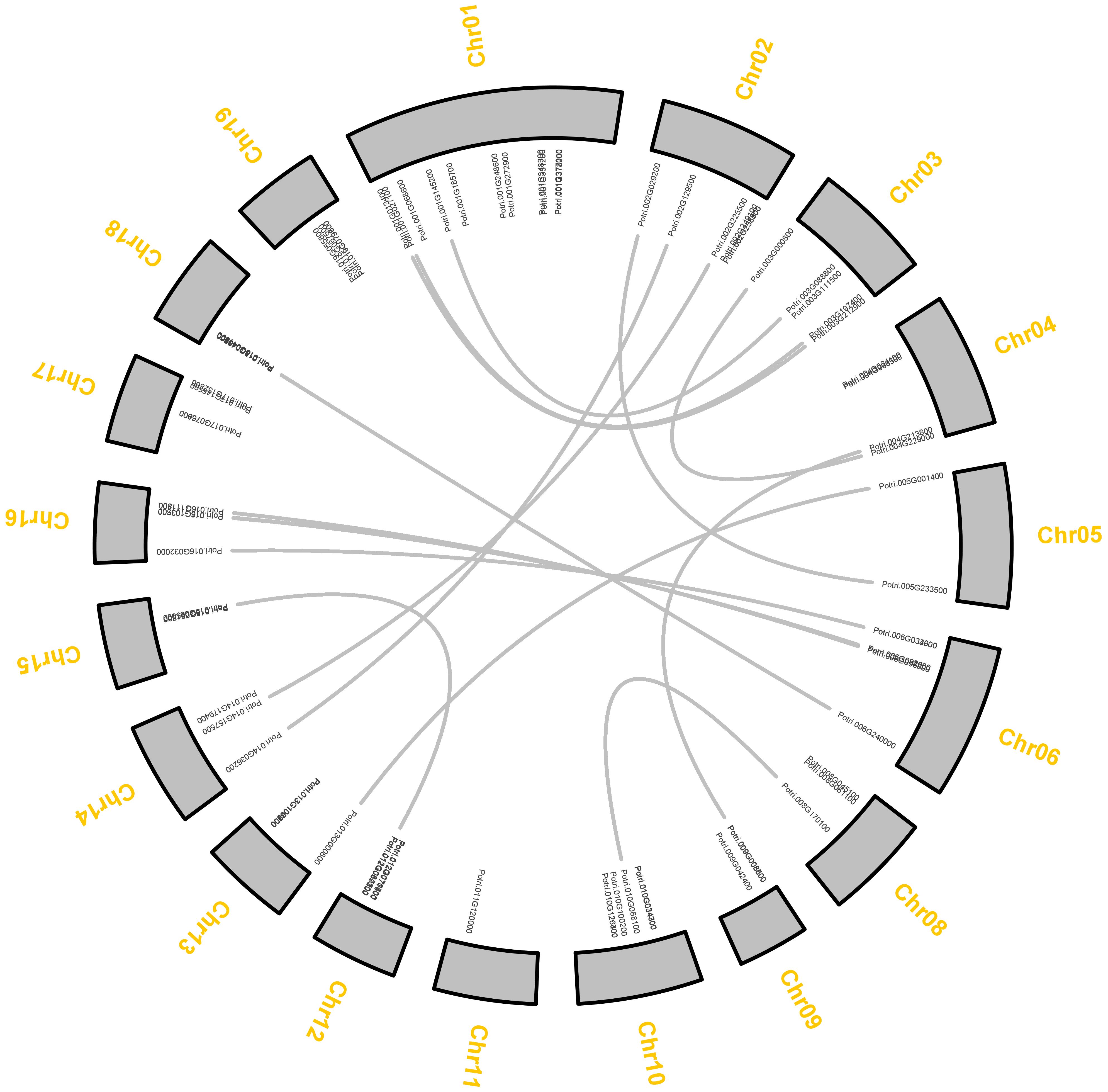


Supplementary Figure S1. Collinearity analysis of genes within poplar. Each colored bar represents a chromosome as indicated. Gene IDs are labeled on the basis of their positions on the chromosomes. The ends of the lines point toward paralog pairs derived from segmental duplication.


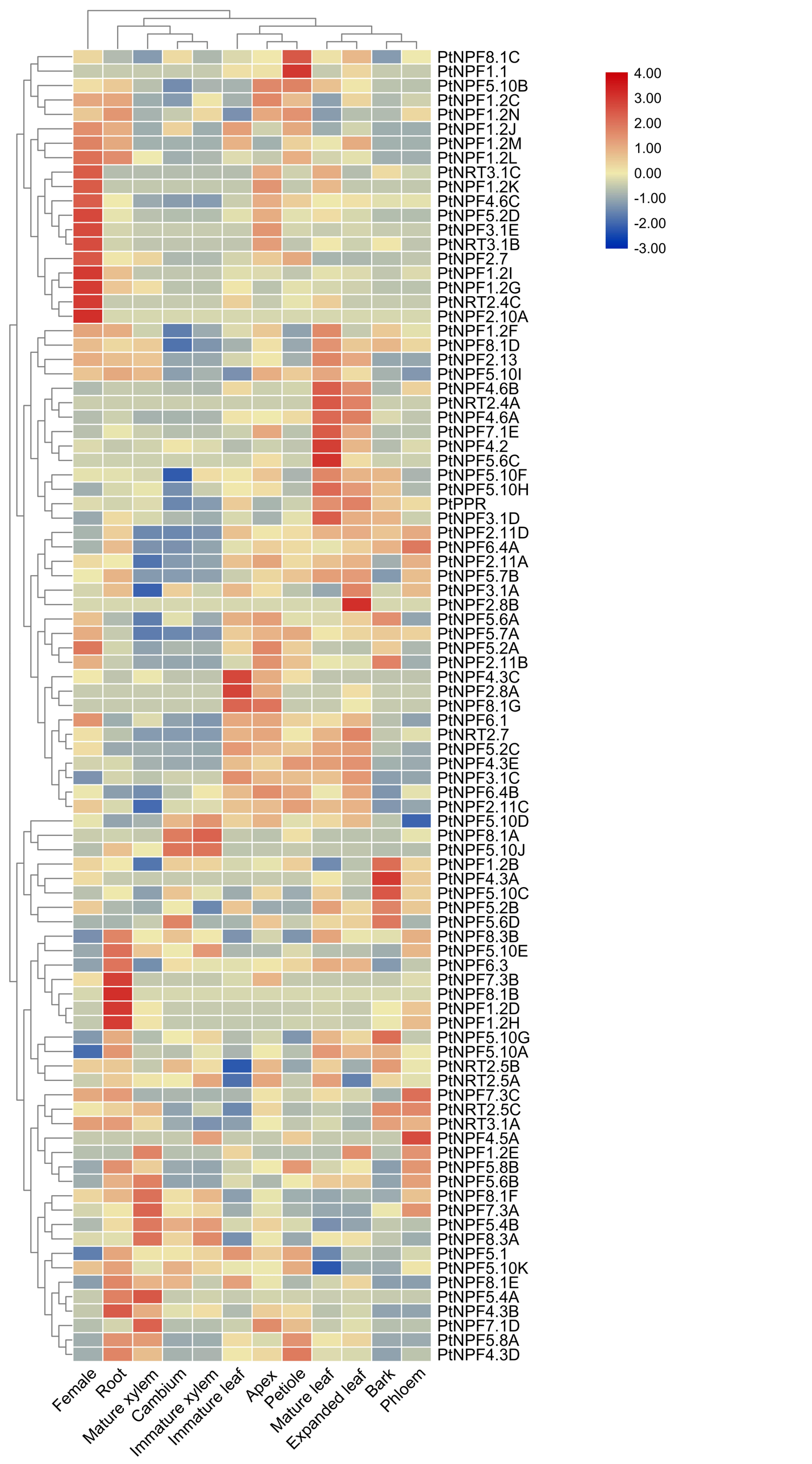


Supplementary Figure S2. Expression profiles of NRT family genes in P. tomentosa. Heatmap of tissue-specific expression.


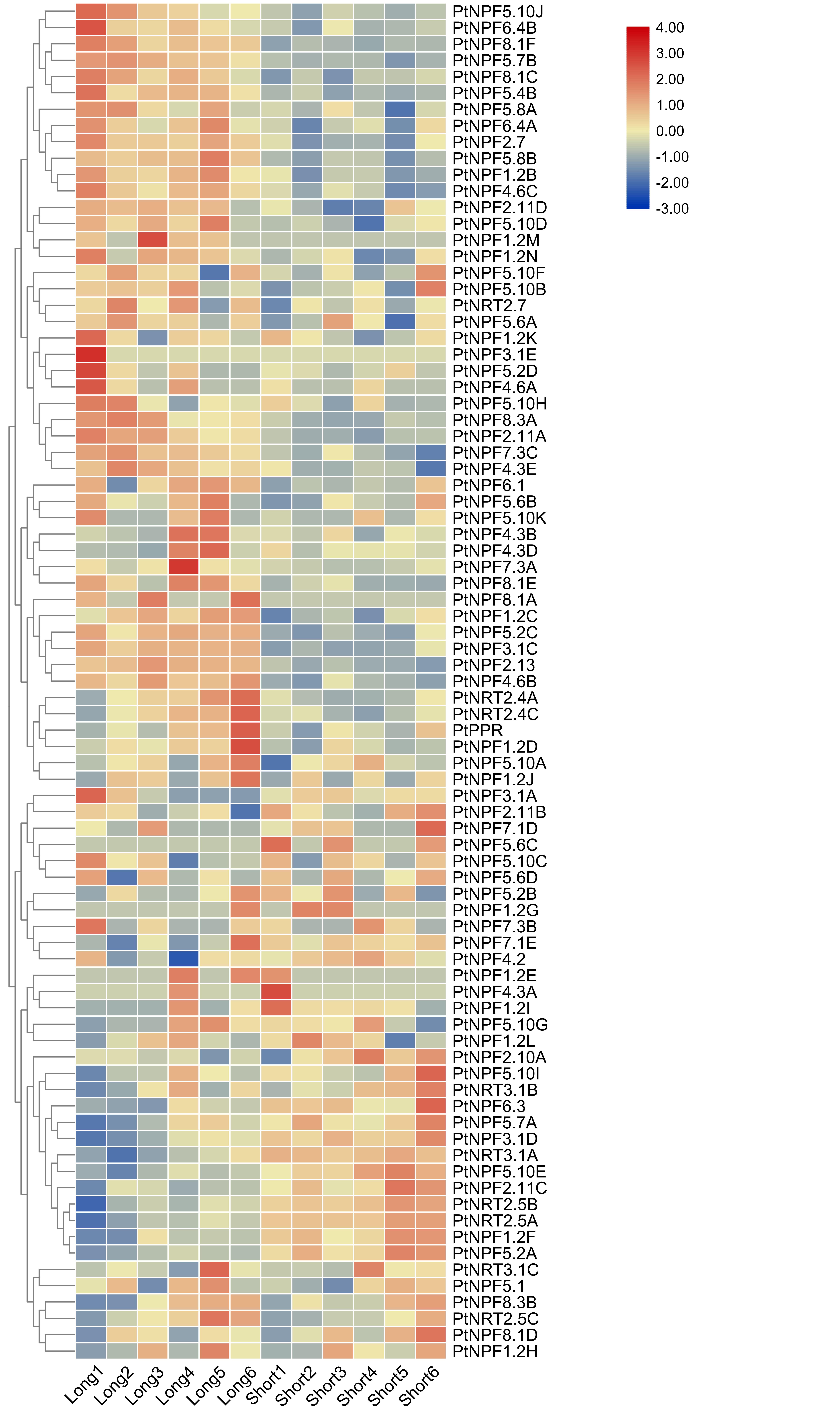


Supplementary Figure S3. Expression profiles of NRT family genes in P. tomentosa. Long-shoot leaves and short branch leaves. Long: annual long-shoot leaves; Short: perennial short-branch leaves. The development of leaves, showing gradual maturation from 1 to 6.


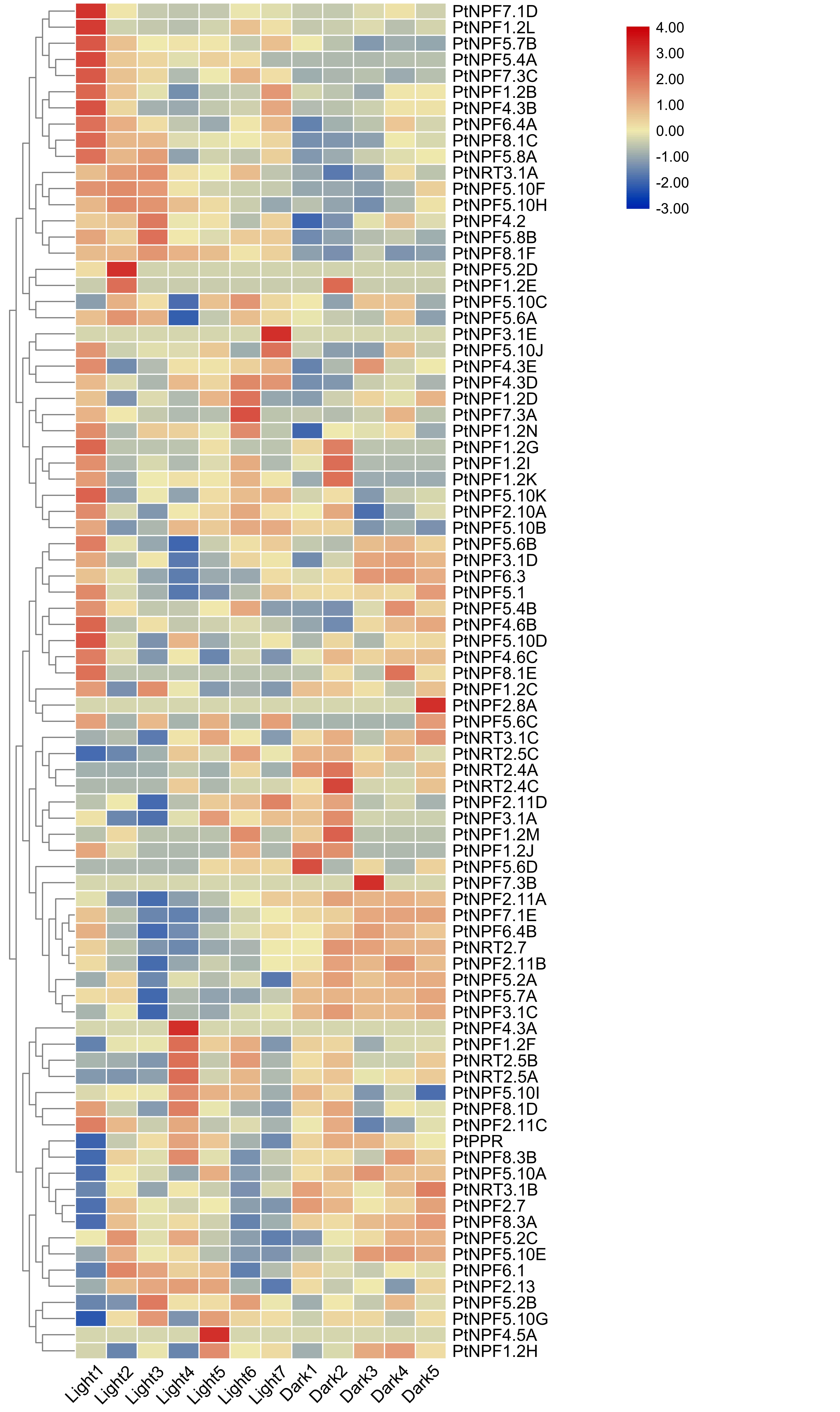


Supplementary Figure S4. Expression profiles of NRT family genes in P. tomentosa. Heatmap of circadian rhythm-related expression. Samples were collected every two hours, under a cycle of 14 h light and 10 h dark.





Supplementary Figure S5. Expression profiles of NRT family genes in P. tomentosa. Heatmap of stress-induced expression. ABA: ABA treatment; D: Drought; HM: Heavy metal; HS: High salt; HT: High temperature. Labels 1-5 correspond to samples collected at 1, 3, 6, 12, and 18 h, respectively.


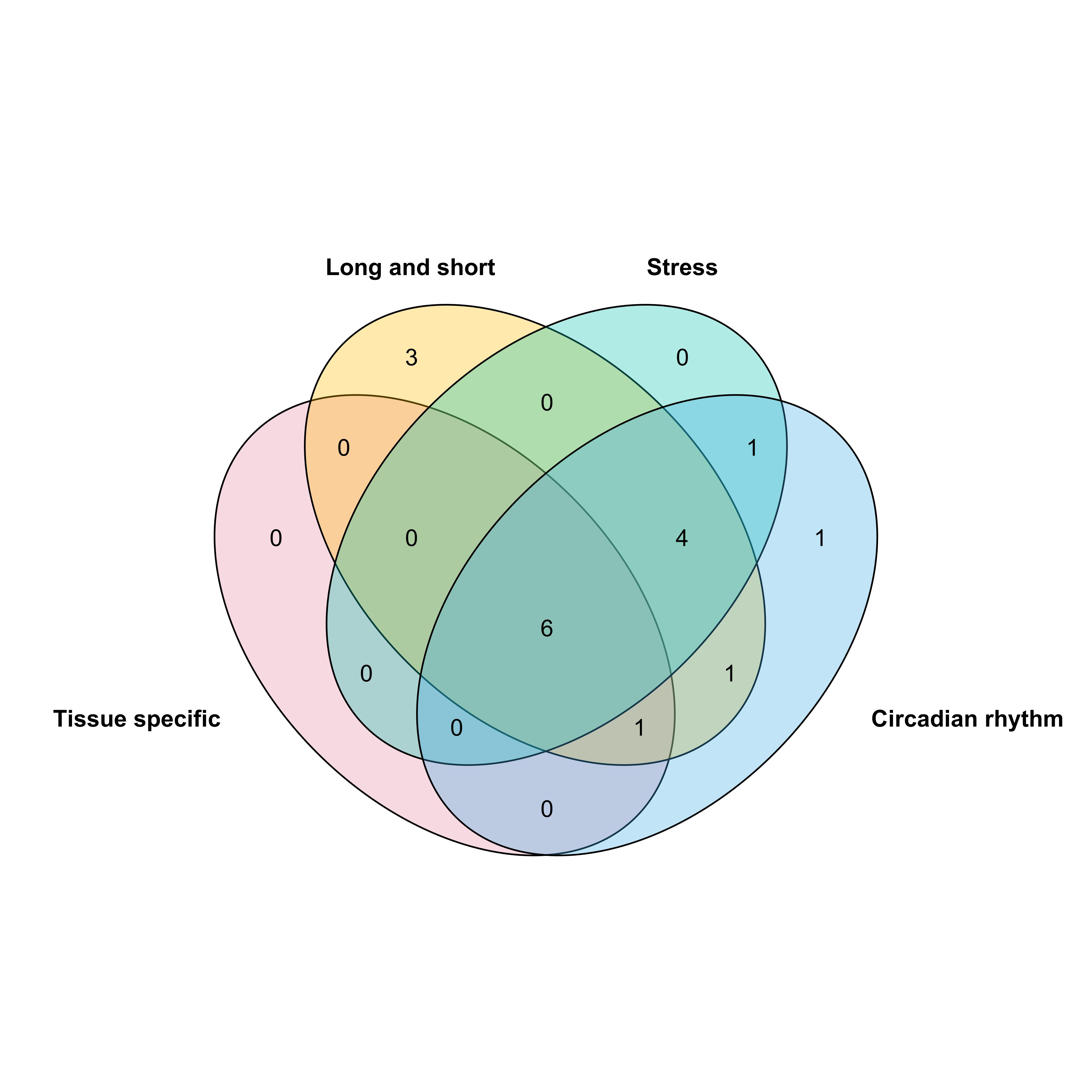


Supplementary Figure S6. Venn diagram of non-expressed genes.


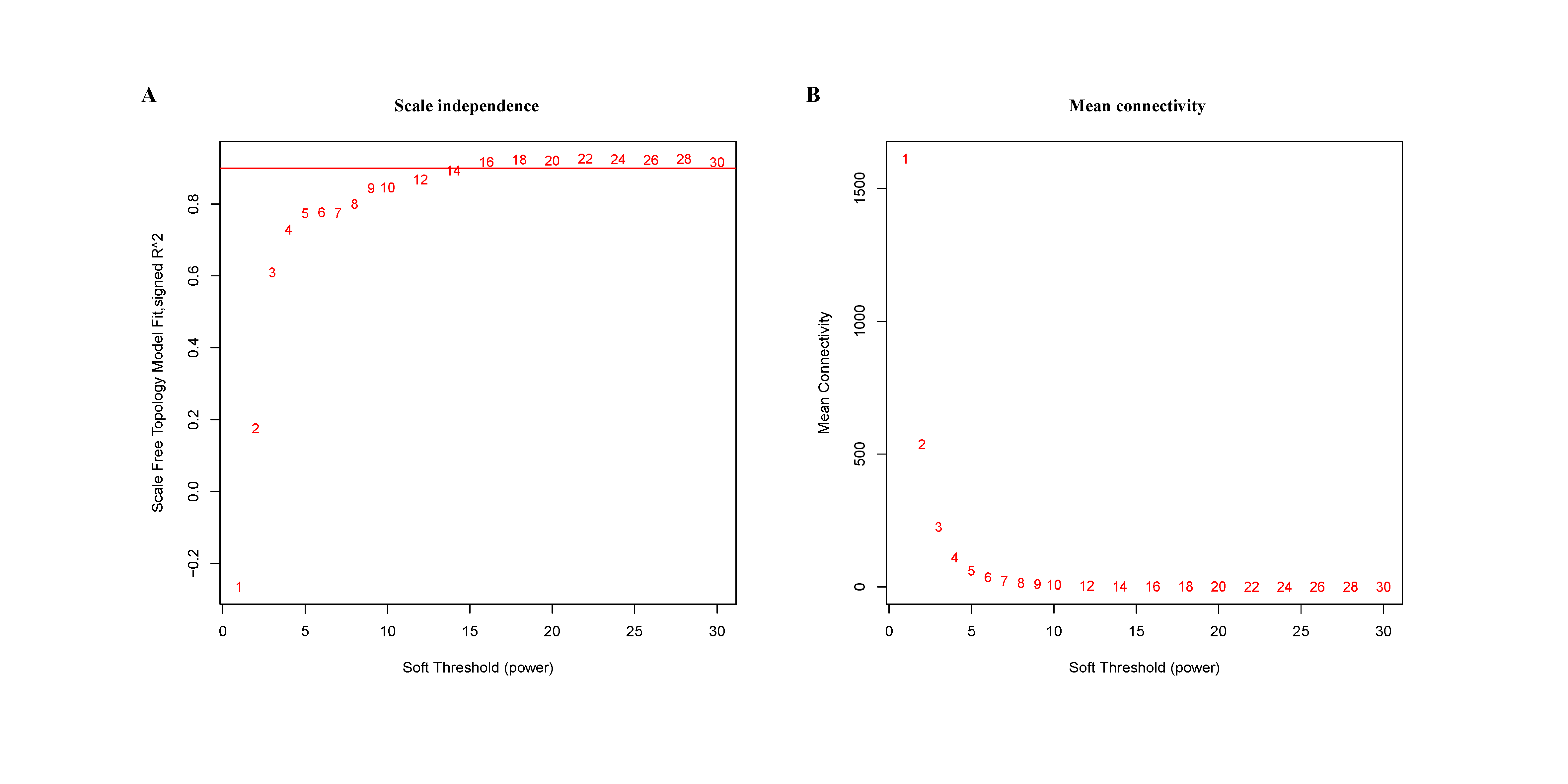


Supplementary Figure S7. Network topologies for various soft-thresholding powers. Numbers in the plots indicate the corresponding soft thresholding powers. The approximate scale-free topology can be attained at the soft-thresholding power of 12.


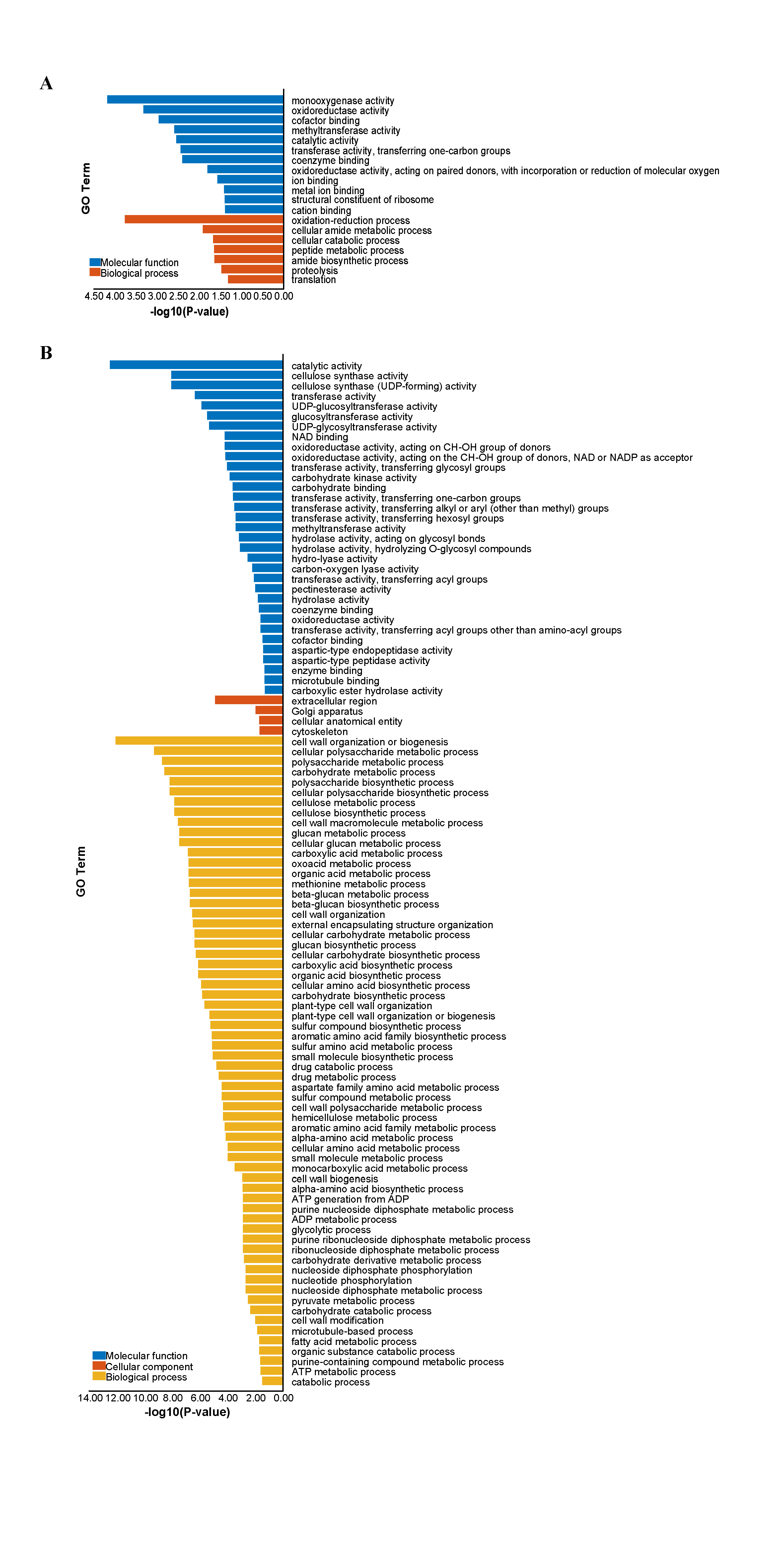


Supplementary Figure S8. GO clustering of black and brown module genes. **A**: black model. **B**: brown model.

**

**

Supplementary Figure S9. *Cis*-regulatory elements (CREs) of the promoters of *NRT* family genes in *P. tomentosa*.
