# Supplementary material for: Genetic Effects and Expression Patterns of the Nitrate Transporter (NRT) Gene Family in Populus tomentosa
Source: Front Plant Sci. 2021 May 13;12:661635. doi: 10.3389/fpls.2021.661635 (PMC8155728; doi:10.3389/fpls.2021.661635)
Supplement: Supplementary file 3 [file Data_Sheet_3.docx]

1. Installation Package

if (!requireNamespace("BiocManager", quietly = TRUE))

install.packages("BiocManager")

BiocManager::install(c("AnnotationDbi", "impute","GO.db", "preprocessCore"))

site="https://mirrors.tuna.tsinghua.edu.cn/CRAN"

install.packages(c("WGCNA", "stringr", "reshape2"), repos=site)

BiocManager::install("BiocParallel")

install.packages("flashClust")

#

http://bioconductor.riken.jp/packages/3.0/data/annotation/html/GO.db.html

install.packages("E:/R-3.6.1/GO.db_3.0.0.zip", repos = NULL, type = "win.binary")

1. Data reading

rm(list = ls())

Expr1 <- read.table("Summer.txt", header = TRUE)

################################## (2) log-transform TPM using log2(x+1)

Expr2 <- log(Expr1+1, 2)

dim(Expr2)

## [1] 25540 90

head(Expr2)[,1:8]

1. Load the package

library(WGCNA) # version 1.63

library(flashClust)

library(reshape2)

library(stringr)

library(BiocParallel)

options(stringsAsFactors = FALSE)

# mutiple Threads

enableWGCNAThreads()

register(MulticoreParam(60))

allowWGCNAThreads(nThreads = 60)

# type of network

##type = "signed"

# correlation method

corType = "pearson"

#another correlation type is “bicor”

# Restricting the number of excluded outliers

# specifies the maximum percentile of data that can be

# considered outliers on either side of the median separately.

maxPOutliers = 0.05

# Dealing with binary data

robustY = FALSE

1. Converted to a matrix of samples in rows, genes in columns

Expr_t <- as.data.frame(t(Expr2))

#Expr_t[1:6,1:6]

1. Detect missing values

gsg = goodSamplesGenes(Expr_t, verbose = 3)

## Flagging genes and samples with too many missing values...

## ..step 1

if (!gsg$allOK){

# Optionally, print the gene and sample names that were removed:

if (sum(!gsg$goodGenes)>0)

printFlush(paste("Removing genes:",

paste(names(Expr2)[!gsg$goodGenes], collapse = ",")));

if (sum(!gsg$goodSamples)>0)

printFlush(paste("Removing samples:",

paste(rownames(Expr2)[!gsg$goodSamples], collapse = ",")));

# Remove the offending genes and samples from the data:

Expr_t = Expr_t[gsg$goodSamples, gsg$goodGenes]

}

nGenes = ncol(Expr_t)

nSamples = nrow(Expr_t)

dim(Expr_t)

## [1] 90 25540

1. Soft threshold filtering

#################################### (5) Check for outliers

sampleTree <- hclust(dist(Expr_t), method = "average")

par(mar = c(0,4,2,0))

pdf(file = "sampleTree.pdf", width = 12, height = 9)

plot(sampleTree, main = "Sample clustering to detect outliers", sub="", xlab="")

dev.off()

clust <- cutreeStatic(sampleTree, cutHeight = 320, minSize = 8)

# outlier samples

rownames(Expr_t)[clust==0]

#[1] "A0_5GR" "E2_6GP" "E2_5GR" "E2_6GR" "C9_5GR_1"

# remove outlier samples

keepSamples <- (clust != 0)

Expr3 <- Expr_t[keepSamples, ]

# the number of samples and genes

dim(Expr3)

#[1] 90 25540

geneNames <- colnames(Expr3)

################################## (6) choose soft threshold "beta"

# choosing a set of soft-thresholding powers

powers <- c(seq(1, 10, by=1), seq(12, 30, by=2))

type ="unsigned" ##adjacency = |cor|^power

# call network topology analysis function

# type = "signed": adjacency = (0.5 * (1+cor) )^power;

# type = "signed hybrid": adjacency = cor^power if cor>0 and 0 otherwise;

# type = "distance": adjacency = (1-(dist/max(dist))^2)^power.

#################sft <- pickSoftThreshold(Expr3, powerVector = powers,

networkType = type, corFnc = corType,

corOptions = list(use = 'p', maxPOutliers = maxPOutliers),

verbose=5)

sft = pickSoftThreshold(Expr3, powerVector=powers,

networkType=type, verbose=5)

# plot the results

## Scale-free topology fit index as a function of the soft-thresholding power

##From tutorial figure legend: https://labs.genetics.ucla.edu/horvath/CoexpressionNetwork/Rpackages/WGCNA/Tutorials/FemaleLiver-02-networkConstr-blockwise.pdf

## Analysis of network topology for various soft-thresholding powers. The left

## panel shows the scale-free fit index (y-axis) as a function of the soft-

## thresholding power (x-axis). The right panel displays the mean connectivity

## (degree, y-axis) as a function of the soft-thresholding power (x-axis)

sizeGrWindow(9,5)

par(mfrow= c(1,2))

cex1=0.9

pdf("wgcna_soft.thresholding.pdf")

# R2 ~ soft-thresholding power

plot(sft$fitIndices[,1], -sign(sft$fitIndices[,3])*sft$fitIndices[,2],

xlab="Soft Threshold (power)",ylab="Scale Free Topology Model Fit,signed R^2",type="n",

main = paste("Scale independence"))

text(sft$fitIndices[,1], -sign(sft$fitIndices[,3])*sft$fitIndices[,2],

labels=powers,cex=cex1,col="red")

# this line corresponds to using an R^2 cut-off of h

abline(h=0.9,col="red")

# Mean connectivity ~ soft-thresholding power

plot(sft$fitIndices[,1], sft$fitIndices[,5],

xlab="Soft Threshold (power)",ylab="Mean Connectivity", type="n",

main = paste("Mean connectivity"))

text(sft$fitIndices[,1], sft$fitIndices[,5], labels=powers, cex=cex1,col="red")

dev.off()

power = sft$powerEstimate

power

## [1] 12

1. Network construction

############################## (7) Generating adjacency and TOM similarity matrices based on the selected softpower and detect Modules

softPower <- 12 ##### The predicted value of the previous step ## [1] 12

net <- blockwiseModules(

Expr3,

maxBlockSize = dim(Expr3)[2],

corType = corType,

# calclute the adjacency matrix

power = 7,

networkType = type,

# TOM similarity matrices

TOMType = type,

saveTOMs = TRUE,

saveTOMFileBase = "blockwiseTOM",

# Module identification using dynamic tree cut

#deepSplit = 4,

minModuleSize = 30,

mergeCutHeight =0.25,

numericLabels = F, # modudule named in number

nThreads = 0,

verbose = 3

)

# Module merging options

net = blockwiseModules(Expr3, power = power, maxBlockSize = nGenes,

TOMType = type, minModuleSize = 30,

reassignThreshold = 0, mergeCutHeight = 0.25,

numericLabels = TRUE, pamRespectsDendro = FALSE,

saveTOMs=TRUE, corType = corType,

maxPOutliers=maxPOutliers, loadTOMs=TRUE,

saveTOMFileBase = "blockwiseTOM1111",

verbose = 3)

## Calculating module eigengenes block-wise from all genes

## Flagging genes and samples with too many missing values...

## ..step 1

## ..Working on block 1 .

## TOM calculation: adjacency..

## ..will use 47 parallel threads.

## Fraction of slow calculations: 0.000000

## ..connectivity..

## ..matrix multiplication (system BLAS)..

## ..normalization..

## ..done.

## ..saving TOM for block 1 into file LiverFemaleClean.txt.tom-block.1.RData

## ....clustering..

## ....detecting modules..

## ....calculating module eigengenes..

## ....checking kME in modules..

## ..removing 3 genes from module 1 because their KME is too low.

## ..removing 5 genes from module 12 because their KME is too low.

## ..removing 1 genes from module 14 because their KME is too low.

## ..merging modules that are too close..

## mergeCloseModules: Merging modules whose distance is less than 0.25

## Calculating new MEs...

table(net$colors)

1. Hierarchical cluster tree displays each module

moduleLabels = net$colors

moduleColors = labels2colors(moduleLabels)

sizeGrWindow(12,9)

par(cex = 0.6)

par(mar = c(0,4,2,0))

pdf("plotDendroAndColors222.pdf", width = 12, height = 8)

plotDendroAndColors(net$dendrograms[[1]], moduleColors[net$blockGenes[[1]]],

"Module colors",

dendroLabels = FALSE, hang = 0.03,

addGuide = TRUE, guideHang = 0.05)

dev.off()

save(Expr3, sft, net, moduleColors, file = "wgcna-network.Rdata")

1. Draw the correlation diagram between modules

MEs = net$MEs

MEs = moduleEigengenes(Expr3, moduleColors)$eigengenes

MET = orderMEs(MEs)

sizeGrWindow(7, 6)

pdf("module_correlation333.pdf")

plotEigengeneNetworks(MET, "Eigengene adjacency heatmap",

marHeatmap = c(3,4,2,2), plotDendrograms = FALSE,

xLabelsAngle = 90)

dev.off()

# The correlation diagram of each module obtained by the clustering of intergene expressions includes the tree diagram

plotEigengeneNetworks(MET, "Eigengene adjacency heatmap",

marDendro = c(3,3,2,4),

marHeatmap = c(3,4,2,2), plotDendrograms = T,

xLabelsAngle = 90)

1. Visualize gene networks (TOM plot)

## load("blockwiseTOM-block.1.RData")

## Loading objects:

## TOM

load(net$TOMFiles[1], verbose=T)

TOM <- as.matrix(TOM)

dissTOM = 1-TOM

# Transform dissTOM with a power to make moderately strong

# connections more visible in the heatmap

plotTOM = dissTOM^7

# Set diagonal to NA for a nicer plot

diag(plotTOM) = NA

# Call the plot function

sizeGrWindow(12,9)

pdf(file="TOM.pdf", width = 10, height = 6)

TOMplot(plotTOM, net$dendrograms, moduleColors,

main = "Network heatmap plot, all genes,mc.cores=6")

dev.off()

1. Correlation between traits and modules

sizeGrWindow(12,9)

par(cex = 0.6)

par(mar = c(0,4,2,0))

trait=read.table("Summer.pheno.txt",head=TRUE)

pdf("phenotype_trait222.pdf", width = 15, height = 12)

plotEigengeneNetworks(Expr1,

"Eigengene adjacency heatmap",

marDendro = c(3,3,2,4), marHeatmap = c(3,4,2,2), plotDendrograms = T, xLabelsAngle = 90)

dev.off()

1. Modules are associated with phenotypic data

trait=read.table("Summer.pheno.txt",head=T)

if (corType=="pearson") {

modTraitCor = cor(MET, trait, use = "p")

modTraitP = corPvalueStudent(modTraitCor, nSamples)

} else {

modTraitCorP = bicorAndPvalue(MET, trait, robustY=robustY)

modTraitCor = modTraitCorP$bicor

modTraitP = modTraitCorP$p

}

textMatrix = paste(signif(modTraitCor, 2), "\n(", signif(modTraitP, 1), ")", sep = "")

dim(textMatrix) = dim(modTraitCor)

pdf("modual_correlation22221111.pdf", width = 4, height = 12)

labeledHeatmap(Matrix = modTraitCor, xLabels = colnames(trait),

yLabels = colnames(MET),

cex.lab = 0.5,

ySymbols = colnames(MET), colorLabels = FALSE,

colors = blueWhiteRed(50),

textMatrix = textMatrix , setStdMargins = FALSE,

cex.text = 0.5, zlim = c(-1,1),

main = paste("Module-trait relationships"))

dev.off()

1. Calculate the correlation matrix between modules and genes

if (corType=="pearson") {

geneModuleMembership = as.data.frame(cor(Expr3, MET, use = "p"))

MMPvalue = as.data.frame(corPvalueStudent(

as.matrix(geneModuleMembership), nSamples))

} else {

geneModuleMembershipA = pearsonAndPvalue(Expr3, MET, robustY=robustY)

geneModuleMembership = geneModuleMembershipA$pearson

MMPvalue = geneModuleMembershipA$p

}

1. Calculate the correlation matrix between traits and genes

if (corType=="pearson") {

geneTraitCor = as.data.frame(cor(Expr3, trait, use = "p"))

geneTraitP = as.data.frame(corPvalueStudent(as.matrix(geneTraitCor), nSamples))

} else {

geneTraitCorA = pearsonAndPvalue(Expr3, trait, robustY=robustY)

geneTraitCor = as.data.frame(geneTraitCorA$pearson)

geneTraitP = as.data.frame(geneTraitCorA$p)

}

## Warning in pearson(x, y, use = use, ...): pearson: zero MAD in variable 'y'.

## Pearson correlation was used for individual columns with zero (or missing)

## MAD.

1. Finally, combine the two correlation matrices and specify the modules of interest for analysis

module = "brown" # no writing ME

pheno = "Autumn"

modNames = substring(colnames(MET), 3)

module_column = match(module, modNames)

pheno_column = match(pheno,colnames(trait))

moduleGenes = moduleColors == module

sizeGrWindow(7, 7)

par(mfrow = c(1,1))

pdf("Module _gene correlation.pdf")

verboseScatterplot(abs(geneModuleMembership[moduleGenes, module_column]),

abs(geneTraitCor[moduleGenes, pheno_column]),

xlab = paste("Module Membership in", module, "module"),

ylab = paste("Gene significance for", pheno),

main = paste("Module membership vs. gene significance\n"),

cex.main = 1.2, cex.lab = 1.2, cex.axis = 1.2, col = module)

dev.off()

1. Extract module

module_colors <- setdiff(unique(moduleColors), "grey")

for (color in module_colors){module <- geneNames[which(moduleColors==color)]

write.table(module, paste("module_",color, ".txt",sep=""), sep="\t", row.names=FALSE, col.names=FALSE,quote=FALSE) }

# Export the network into edge and node list files Cytoscape can read

load(net$TOMFiles[1], verbose=T)

TOM <- as.matrix(TOM)

dimnames(TOM) <- list(geneNames, geneNames)

# The module to visualize

for(i in module_colors){

modules = i

# The gene to visualize

probes = colnames(Expr3)

inModule = is.finite(match(moduleColors, modules))

modProbes = probes[inModule]

# Candidate gene TOM

modTOM = TOM[inModule, inModule]

dimnames(modTOM) = list(modProbes, modProbes)

# Export the network into edge and node list files Cytoscape can read

cyt = exportNetworkToCytoscape(modTOM,edgeFile = paste("cyt_edges_", paste(modules, collapse="-"), ".txt", sep=""),nodeFile=paste("cyt_nodes_", paste(modules, collapse="-"), ".txt", sep=""),weighted = TRUE,threshold = 0.3, nodeNames = modProbes, nodeAttr = moduleColors[inModule]) }

1. Screening hub genes

datME=moduleEigengenes(Expr3,moduleColors,trapErrors=FALSE)$eigengens

NS1=networkScreening(datTraits$Tum_Gra,datME,Expr3,oddPower=3,blockSize=1000,minimumSampleSize=4,addMEy=TRUE,removeDiag=FALSE,weightESy=0.5)

GeneResultsNetworkScreening=data.frame(GeneName=row.names(NS1),NS1)

write.table(GeneResultsNetworkScreening,file=“GeneResultsNetworkScreening for hubgene.csv”,row.names=F,sep=“,”)

################# The heat map shows the hub gene。

datME=moduleEigengenes(Expr3,moduleColors,trapErrors=FALSE)$eigengens

sizeGrWindow(15,15)

topList=rank(NS1$p.Weighted,ties,method=“first”)<=30

gene.names=names(Expr3[topList]
