# Supplementary material for: Genetic Effects and Expression Patterns of the Nitrate Transporter (NRT) Gene Family in Populus tomentosa
Source: Front Plant Sci. 2021 May 13;12:661635. doi: 10.3389/fpls.2021.661635 (PMC8155728; doi:10.3389/fpls.2021.661635)
Supplement: Supplementary file 4 [file Data_Sheet_4.docx]

# Convert SNP to 0, 1, 2 format

plink --file myplink89 --chr-set 30 --geno 0.1 --mind 0.1 --maf 0.01 --hwe 1e-6 --recodeA --out SNP89

awk '{$2="";$3="";$4="";$5="";$6="";print $0}' SNP89.raw > snp89.txt

# The ranks of transpose

awk '{i=1;while(i <= NF){col[i]=col[i] $i " ";i=i+1}} END {i=1;while(i<=NF){print col[i];i=i+1}}' snp89.txt | sed 's/[ \t]*$//g' > ptosnp89.txt

install.packages('MatrixEQTL')#eQTL分析

library(MatrixEQTL)

1. Data

Setting of early data files and relevant parameters：

#SNP data

SNP_file_name = paste0("snp.txt", sep="")

snps_location_file_name = paste0("ptosnploc.txt", sep="")

# Gene expression data

expression_file_name = paste0("GE.txt", sep="")

gene_location_file_name = paste0("geneloc.txt", sep="")

# The phenotypic data

# Set to character() for no covariates

covariates_file_name = paste0("autCovariates.txt", sep="")

# The output file

output_file_name_cis = tempfile()

output_file_name_tra = tempfile()

# Association level threshold

pvOutputThreshold_cis = 2e-4

pvOutputThreshold_tra = 1e-4

# Error covariance data storage

# Set to numeric() for identity

errorCovariance = numeric()

# errorCovariance =read.table("Sample_Data/errorCovariance.txt")

# Gene-snp distance threshold

cisDist = 2.5e5

#Model selection. modelANOVA, modelLINEAR or modelLINEAR_CROSS。

useModel = modelLINEAR

## SNP data structure construction

snps = SlicedData$new()

snps$fileDelimiter = "\t" # the space character

snps$fileOmitCharacters = "NA" #denote missing values

snps$fileSkipRows = 1 # one row of column labels

snps$fileSkipColumns = 1 # one column of row labels

snps$fileSliceSize = 2000 # read file in slices of 2,000 rows

snps$LoadFile(SNP_file_name)

## Gene expression data construction

gene = SlicedData$new()

gene$fileDelimiter = "\t" # the TAB character

gene$fileOmitCharacters = "NA" #denote missing values

gene$fileSkipRows = 1 # one row of column labels

gene$fileSkipColumns = 1 # one column of row labels

gene$fileSliceSize = 2000 # read file in slices of 2,000 rows

gene$LoadFile(expression_file_name)

## The phenotypic data

cvrt = SlicedData$new()

cvrt$fileDelimiter = "\t" # the TAB character

cvrt$fileOmitCharacters = "NA" #denote missing values

cvrt$fileSkipRows = 1 # one row of column labels

cvrt$fileSkipColumns = 1 # one column of row labels

if(length(covariates_file_name)>0) {

cvrt$LoadFile(covariates_file_name)

}

## Gene and SNP location information

snpspos =read.table(snps_location_file_name, header = TRUE, stringsAsFactors = FALSE)

genepos =read.table(gene_location_file_name, header = TRUE, stringsAsFactors = FALSE)

2. The data analysis

me = Matrix_eQTL_main(

snps = snps,

gene = gene,

cvrt = cvrt,

output_file_name =output_file_name_tra,

pvOutputThreshold =pvOutputThreshold_tra,

useModel = useModel,

errorCovariance = errorCovariance,

verbose = TRUE,

output_file_name.cis =output_file_name_cis,

pvOutputThreshold.cis =pvOutputThreshold_cis,

snpspos = snpspos,

genepos = genepos,

cisDist = cisDist,

pvalue.hist = "qqplot",

min.pv.by.genesnp = FALSE,

noFDRsaveMemory= FALSE);

unlink(output_file_name_tra)

unlink(output_file_name_cis)

## Results:

write.table(me$cis$eqtls, "autcis.txt")

write.table(me$trans$eqtls, "auttrans.txt")

tar -jcvf d.tar.bz2 auttrans.txt

## Plot the Q-Q plot of local and distant p-values

pdf(file = "autQQplot.pdf", width = 10, height = 8)

plot(me, pch = 16, cex = 0.7)

dev.off()

install.packages(“qqman”)

vignette('qqman')

library(qqman)

A <- read.table("sum_cis_man.txt", header = T)

pdf(file = "sum_cis.pdf", width=10, height=5)

manhattan(A, main = "Manhattan Plot", ylim = c(0, 30), cex = 0.6, cex.axis = 0.9, col = c("blue4", "orange3"), annotateTop = FALSE, suggestiveline = F, genomewideline = -log10(1e-20))

dev.off()
